# A Versatile Approach to Access Trimetallic Complexes Based on Trisphosphinite Ligands

**DOI:** 10.3390/molecules25030593

**Published:** 2020-01-29

**Authors:** Juan Miranda-Pizarro, Macarena G. Alférez, M. Dolores Fernández-Martínez, Eleuterio Álvarez, Celia Maya, Jesús Campos

**Affiliations:** Instituto de Investigaciones Químicas (IIQ), Departamento de Química Inorgánica and Centro de Innovación en Química Avanzada (ORFEO-CINQA), Consejo Superior de Investigaciones Científicas (CSIC) and University of Sevilla, Avenida Américo Vespucio 49, 41092 Sevilla, Spain; juan.miranda@iiq.csic.es (J.M.-P.); macarena.gonzalez@iiq.csic.es (M.G.A.); mariadolores.fernandez@urv.cat (M.D.F.-M.); ealvarez@iiq.csic.es (E.Á.); maya@us.es (C.M.)

**Keywords:** phosphinite, multidentate ligands, polymetallic, supramolecular chemistry, trimetallic, coordination polymer

## Abstract

A straightforward method for the preparation of trisphosphinite ligands in one step, using only commercially available reagents (1,1,1-tris(4-hydroxyphenyl)ethane and chlorophosphines) is described. We have made use of this approach to prepare a small family of four trisphosphinite ligands of formula [CH_3_C{(C_6_H_4_OR_2_)_3_], where R stands for Ph (**1a**), Xyl (**1b**, Xyl = 2,6-Me_2_-C_6_H_3_), *^i^*Pr (**1c**), and Cy (**1d**). These polyfunctional phosphinites allowed us to investigate their coordination chemistry towards a range of late transition metal precursors. As such, we report here the isolation and full characterization of a number of Au(I), Ag(I), Cu(I), Ir(III), Rh(III) and Ru(II) homotrimetallic complexes, including the structural characterization by X-ray diffraction studies of six of these compounds. We have observed that the flexibility of these trisphosphinites enables a variety of conformations for the different trimetallic species.

## 1. Introduction

The design of new classes of multidentate ligands has attracted much attention over the previous decades in the context of supramolecular chemistry. Owing to the rigidity and predictable geometries adopted by nitrogen and oxygen donors, those have been primarily selected to construct well-defined self-assembled architectures relying on metal-ligand coordination [1,2,3,4,5]. In contrast, multidentate phosphines have been explored to a lesser extent, despite the prominent position that their monovalent versions occupy in organometallic chemistry and homogeneous catalysis. Regardless of their more limited use, the unique features that polydentate phosphines offer (i.e., superior flexibility, irregular topologies, chirality, ^31^P NMR analysis), have led to a rich variety of supramolecular structures derived from metal coordination [6], including cages [7,8,9,10], porous solids [11,12], helicates [13,14], coordination polymers [15,16,17], and even a chiral nanocluster [18]. Besides, many of these polymetallic structures reveal a remarkable capacity for molecular recognition and sensing [19,20], interesting photochemical properties [21,22,23,24], or catalytic applications [25].

With all this in mind, it is somehow surprising that related multidentate phosphinites have barely been investigated. Phosphinites (PR_2_(OR)) were identified as valuable ligands long after the widespread use of phosphines. Their superior *π*-acceptor ability confers the resulting metal complexes distinctive electronic features that have been broadly exploited for catalytic applications [26,27]. Besides, the reduced σ-donating capacity of phosphinites compared to phosphines may facilitate accessing well-defined supramolecular structures in a more selective manner by dynamic coordination-dissociation behavior in solution. In addition, multidentate phosphinites could be further exploited for anchoring homogeneous catalysts to oxide surfaces [28,29] by one or several of their PR_2_(OR) functionalities. The use of polypodal phosphorus ligands is particularly appealing in this context [30].

To the best of our knowledge, the limited number of multidentate phosphinite systems capable of binding more than two metals [31] mostly rely on the chemistry of functionalized calixarenes [32,33,34,35], with clear catalytic potential [36]. In a more recent study, a triruthenium complex constructed around a tridentate aminophosphine–phosphinite ligand was recognized as an efficient homogenous catalyst in transfer hydrogenation reactions [37]. To better understand the chemistry of these underdeveloped systems, we decided to synthesize several homotrimetallic complexes stabilized by a family of tridentate phosphinites. An additional advantage of phosphinites, compared to more traditional phosphines, is the easiness of the experimental procedures by which these ligands can be prepared. Polydentate phosphines are laborious to synthesize, often lack selectivity, and require the use of highly reactive reagents [9,38,39,40,41,42]. In stark contrast, we report herein a simple and versatile method to prepare several tridentate phosphinites by common procedures [43], in one-step, and from commercially available reagents. The coordination chemistry of these ligands, with a range of transition metal precursors, is also described.

## 2. Results and Discussion

### 2.1. Synthesis of Tripodal Phosphinites

**Scheme 1:**
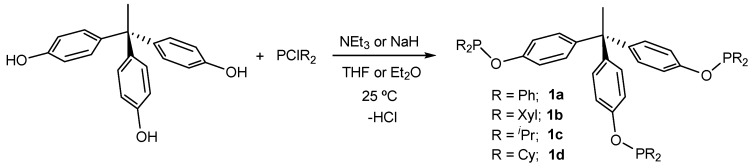
Synthesis of trisphosphinite ligands from 1,1,1-tris(4-hydroxyphenyl)ethane and dihalophosphines.

**Figure 1:**
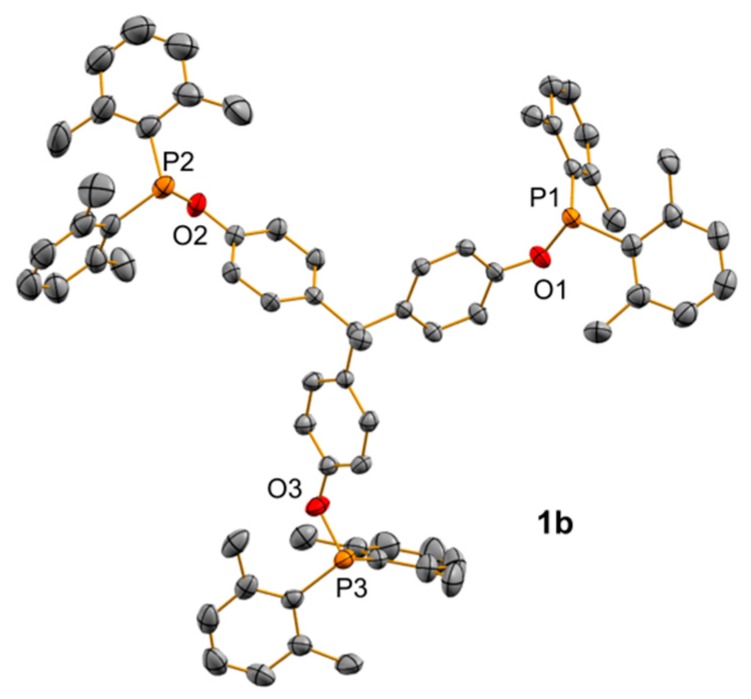
ORTEP view of the solid structure of compound **1b** with thermal ellipsoids set at 50% probability. Hydrogen atoms have been excluded for the sake of clarity.

We have focused on a family of trisphosphinites derived from the condensation of commercially available and affordable 1,1,1-tris(4-hydroxyphenyl)ethane, with several chlorophosphines under basic conditions in good yields (63–84%; Scheme 1). This method contrasts with traditional approaches for the preparation of trisphosphines, which require several synthetic steps and the use of aggressive reagents [9,38,39,40,41,42]. The synthetic procedure is highly versatile and permits the incorporation of phosphines bearing both aliphatic and aromatic substituents, including sterically congested functionalities, such as the two xylyl (2,6-Me_2_-C_6_H_3_) groups per phosphorus center in trisphosphinite **1b**. While trisphosphinites **1a** and **1c** could be readily prepared using triethylamine as the base, after overnight stirring, ligands derived from the bulkier halophosphines PCl(Xyl)_2_ and PClCy_2_ required the use of sodium hydride and longer reaction times. Trisphosphinites **1a**–**d** were fully characterized by multinuclear NMR spectroscopy (see Section 3 and Supplementary Materials for details). A single ^31^P{^1^H} NMR resonance at around 112 ppm was recorded for trisphosphinites **1a** and **1b** bearing aromatic substituents, while it shifted to higher frequencies (*ca.* 146 ppm) for **1c** and **1d**, as expected for phosphinite ligands with aliphatic fragments. To avoid oxidation and increase the overall yield of the trimetallic transition metal complexes, these ligands were typically used after its formation without further purification. However, we managed to grow single crystals of the more sterically congested ligand of the family (**1b**) that were suitable for X-ray diffraction studies. The corresponding ORTEP diagram is depicted in Figure 1 and evinces the *C3* geometry of the trisphosphinite. The P—O bond distances account for around 1.66 Å, while the P—O—C_Ar_ angles are not identical, varying from 117.50(15)° (C28—O2—P2) to 123.18(15)° (C50—O3—P3), though the differences do not seem significant.

### 2.2. Synthesis and Characterization of Trimetallic Complexes

Many of the reported supramolecular structures built over polydentate phosphines contain rigid backbones. The introduction of those rigid cores, such as a phenyl or triazine ring to which the phosphorus centers are directly bound, has permitted the design of trimetallic synthons to construct more complex supramolecular structures in a predictable manner [6]. Besides, the *C3*-symmetry of those systems can be used to attain genuinely chiral cages [44]. The *C3*-trisphosphinites prepared herein introduce an additional degree of structural flexibility that could be an advantage to search for unusual patterns of self-assembly or to access novel supramolecular architectures. However, before entering into that matter, it is necessary to better understand the coordination chemistry of trisphosphinites **1**. To do so, we decided to prepare a series of trimetallic complexes derived from common late transition-metal precursors.

**Scheme 2:**
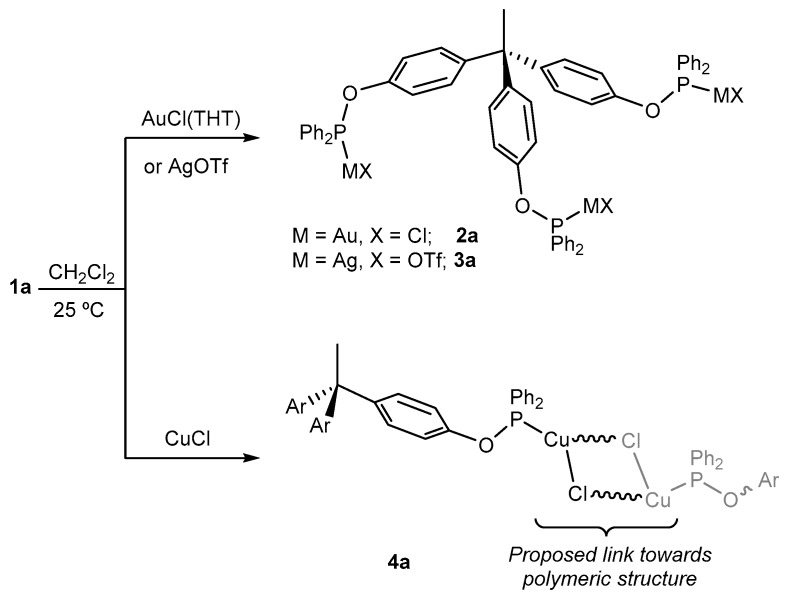
Synthesis of gold (**2a**) and silver (**3a**) trimetallic complexes, and suggested coordination polymer structure of the copper based compound **4a**, based on trisphosphinite **1a**.

**Figure 2:**
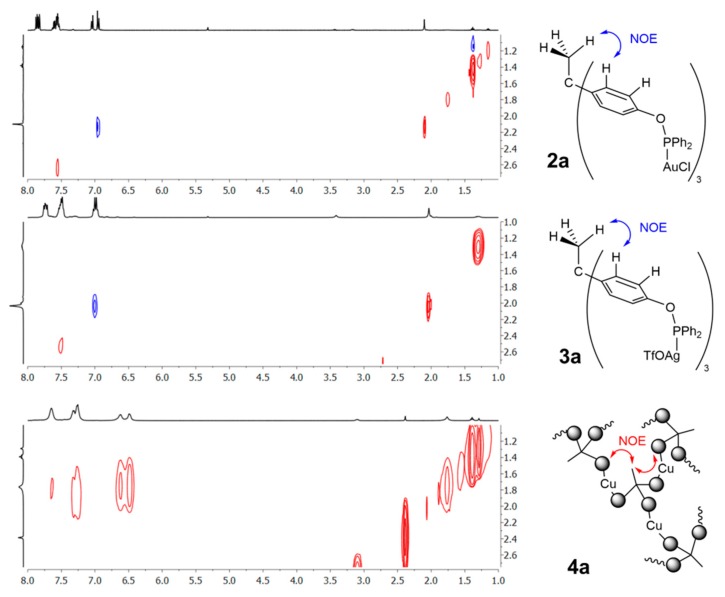
^1^H, ^1^H NOESY experiments of compounds **2a**, **3a,** and **4a**. Grey balls in **4a** represent C_6_H_4_-O-PPh_2_ moieties.

The reaction of **1a** with group 11 metal precursors in a 1:3 molar ratio produced the corresponding trimetallic complexes (Scheme 2). The reaction between **1a** and [AuCl(THT)] (THT = tetrahydrothiophene) in dichloromethane yielded [CH_3_C{(C_6_H_4_OPPh_2_)AuCl}_3_] (**2a**) as an air-stable white powder in excellent isolated yield (95%). Following a similar procedure, the related silver compound [CH_3_C{(C_6_H_4_OPPh_2_)_3_Ag(OTf)}_3_] (**3a**) (OTf^−^ = OSO_2_CF_3_^−^; triflate) was isolated in 72% yield by using AgOTf as the metal precursor. This species was less stable than **2a** under ambient conditions, though it could be handled under air for moderate periods of time. At variance, reaction with CuCl, led to the formation of an insoluble white solid upon concentration of the resulting dichloromethane solution under vacuum. This may result from the formation of a coordination polymer (**4a**) based on the binding of two phosphinite ligands to each of the copper atoms, as previously reported for polydentate phosphines [45,46,47,48], and even for a related bisphosphinite ligand [49]. This solid could be conveniently washed with hexane and dissolved in CDCl_3_ to be characterized by NMR spectroscopy, though subtle variations in temperature or concentration caused the formation of less tractable viscous solutions. Distinctive ^31^P{^1^H} NMR resonances were recorded at 112.8 (**2a**), 120.6 (**3a**), and 96.1 (**4a**) ppm, in agreement with previous related compounds [49,50], while ^1^H NMR spectra did not reveal unusual patterns. A distinctive feature that remains constant along all the compounds studied in this work is the decrease in the scalar coupling constant ^1^*J*_CP_ of the carbon centers directly bound to the phosphorus nuclei upon metal coordination. Thus, while this value accounts for 18 Hz in **1a**, it drastically increases in the corresponding trimetallic species (**2a**, 70 HZ; **3a**, 38 Hz; **4a**, 81 Hz). Interestingly, ^1^H, ^1^H NOESY studies support the proposed polymeric structure of **4a** (Figure 2). As expected, compounds **2a** and **3a** exhibit a single correlation peak of opposite phase to the diagonal signals that connect the apical methyl group of the ligand backbone and one of the aromatic protons of the three *O*-phenyl rings. At variance, compound **4a** reveals several cross peaks that couple the same methyl group with all aromatic signals of the ^1^H NMR spectrum (Figure 2). Moreover, those correlation peaks present the same sign as the diagonal signals [51], altogether supporting the notion of **4a** as a polymeric structure.

**Figure 3:**
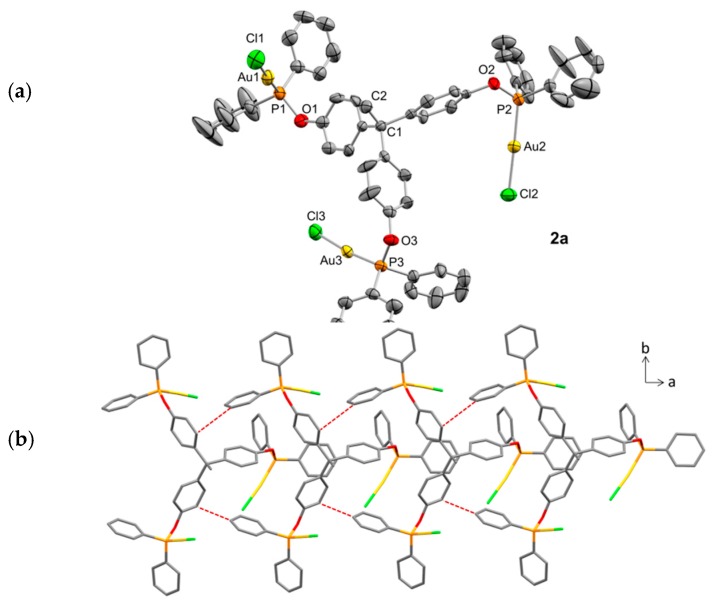
(**a**) ORTEP view of the solid structure of compound **2a** with thermal ellipsoids set at 50% probability. Hydrogen atoms have been excluded for the sake of clarity. Selected bond distances (Å) and angles (°): Au1–P1, 2.214(3); Au1–Cl1, 2.279(4); Au2–P2, 2.228(3); Au2–Cl2, 2.290(3); Au3–P3, 2.219(3); Au3–Cl3, 2.277(3); P1–O1, 1.631(8); P2–O2, 1.621(7); P3–O3, 1.618(8); P1–Au1–Cl1, 177.66(13); P2–Au2–Cl2, 173.51(11); P3–Au3–Cl3, 177.91(11); O1–P1–Au1, 114.0(3); O2–P2–Au2, 111.0(3); O3–P3–Au3, 115.0(3); (**b**) intermolecular weak C—H/π interactions (red dotted lines) that produce 1D supramolecular chains of **2a** along [1 0 0].

The molecular structure of **2a** was further authenticated by X-ray diffraction studies after its crystallization by slow diffusion of pentane over a benzene solution of the compound. Its ORTEP diagram is depicted in Figure 3a. The flexibility of the trisphosphinite is evinced by the different orientations adopted by the Au-Cl termini as a result of the rotation along the C—O and O—P bonds. Thus, the three gold centers appear completely alternate, at variance with the free ligand **1b** (Figure 1), where the orientation of the three lone pairs is convergent [9]. The three P—Au—Cl fragments display normal bond distances, and the expected linear orientation, without any significant distortion (*ca.* 175°). The structure does not contain aurophilic interactions (shortest Au···Au contacts of around 4.28 Å), but the presence of weak C—H/π interactions led to the one-dimensional supramolecular chain along the *a* axis represented in Figure 3b. Each trigold molecule is connected to another two along the *a* direction by two C—H/π interactions characterized by H···C(sp^2^) distances of around 2.74 Å, as well as *d*_H···centroid_ from 3.37 to 3.60 Å; and C—H···centroid angles between 122 and 131°.

**Scheme 3:**
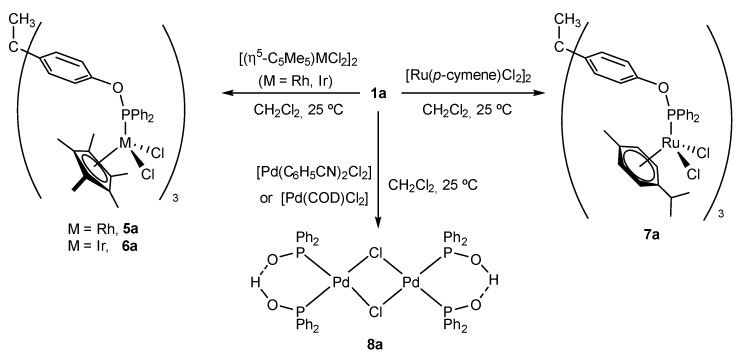
Reaction of trisphosphinite **1a** with several common late transition metal precursors.

To gain further insights into the coordination chemistry of trisphosphinite **1a,** we next focused our attention on other common late transition metal precursors. Reactions of **1a** with [(*η*^5^-C_5_Me_5_)MCl_2_]_2_ (M = Rh, Ir) and [Ru(*p*-cymene)Cl_2_]_2_ produced the corresponding trimetallic compounds [CH_3_C{(C_6_H_4_OPPh_2_)Rh(*η*^5^-C_5_Me_5_)Cl_2_}_3_] (**5a**), [CH_3_C{(C_6_H_4_OPPh_2_)Ir(*η*^5^-C_5_Me_5_)Cl_2_}_3_] (**6a**), and [CH_3_C{(C_6_H_4_OPPh_2_)Ru(*p*-cymene)Cl_2_}_3_] (**7a**) in moderate to good yields (Scheme 3). In contrast, reaction with either [Pt(dmso)_2_Cl_2_] or [Pt(SMe_2_)_2_Cl_2_] did not lead to clean reaction products, whereas precursors [Pd(C_6_H_5_CN)_2_Cl_2_] and [Pd(COD)Cl_2_] (COD = 1,5-cyclooctadiene) yielded **8a** resulting from C—O bond cleavage at trisphenol **1a** in the presence of adventitious water, as previously reported for a related system [49]. 

Coordination of the rhodium and ruthenium precursors to **1a** did not lead to a significant variation of the ^31^P{^1^H} NMR spectra of the resulting complexes, which displays resonances at *ca.* 114 ppm (*c. f.* 110.5 ppm for **1a**). Nevertheless, coordination of the rhodium precursor was evinced by a strong scalar coupling to phosphorus, with the ^31^P{^1^H} signal appearing as a doublet (^1^*J*_PRh_ = 170 Hz) for **5a**. In turn, the ^31^P{^1^H} resonance of the triiridium complex **6a** appeared considerably downshifted at 72.3 ppm. As discussed above, formation of the trimetallic species could be additionally inferred from an increase (*ca.* 30–60 Hz) of the values of the scalar coupling (^1^*J*_CP_) observed for the ^13^C{^1^H} NMR resonances arising from the carbon atoms directly bound to the phosphorus nuclei. Other spectroscopic features can be consulted in the Section 3.

**Figure 4:**
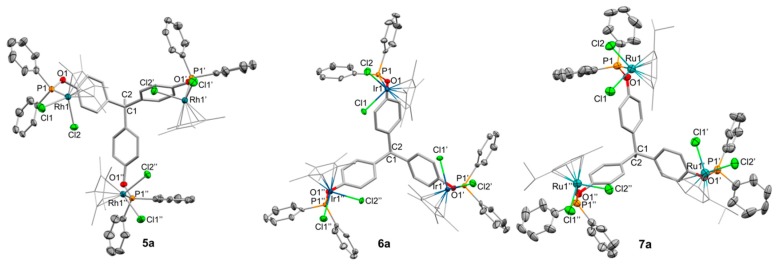
ORTEP view of the solid structure of compounds **5a**, **6a,** and **7a** with thermal ellipsoids set at 50% probability. Hydrogen atoms and solvent molecules have been excluded for the sake of clarity. Selected bond distances (Å) and angles (°): Compound **5a**: Rh1–P1, 2.2829(16); Rh1–Cl1, 2.3945(15); Rh1–Cl2, 2.4017(16); P1–O1, 1.619(4); P1–Rh1–Cl1, 95.44(5); C1–Rh1–Cl2, 155.27(17); P1–Rh1–Cl2, 91.64(6); Cl1–Rh1–Cl2, 88.57(6); Compound **6a**: Ir1–P1, 2.2611(17); Ir1–Cl1, 2.4047(13); Ir1–Cl2, 2.4114(15); P1–O1, 1.625(5); C1–C2, 1.581(14); C1–Ir1–P1, 111.11(18); P1–Ir1–Cl1, 95.92(5); P1–Ir1–Cl2, 92.08(6); O1–P1–Ir1, 115.89(16); Compound **7a** (selected from the two independent molecules in the asymmetric unit): Ru1–P1, 2.305(3); Ru1–Cl2, 2.403(3); Ru1–Cl1, 2.418(3); Ru2–P2, 2.300(3); Ru2–Cl4, 2.410(3); Ru2–Cl3, 2.414(3); P1–O1, 1.637(7); P2–O2, 1.651(7); C2–C1, 1.550(19); P1–Ru1–Cl2, 82.72(9); P1–Ru1–Cl1, 92.94(9); Cl2–Ru1–Cl1, 88.98(10); P2–Ru2–Cl4, 82.69(9); P2–Ru2–Cl3, 93.08(9); Cl4–Ru2–Cl3, 88.89(10); O1–P1–Ru1, 114.7(3); O2–P2–Ru2, 115.3(3).

The molecular formulation of these three complexes was further confirmed by X-ray diffraction studies (Figure 4). Suitable crystals of compounds **5a** and **6a** were grown from slow diffusion of diethyl ether into their dichloromethane solutions at −30 °C, whereas crystals of **7a** were obtained from diffusion of pentane into a dichloromethane/acetone solution of the compound at the same temperature. The *C3*-symmetry of the three complexes is crystallographically maintained, with the three structures grown in trigonal space groups (i.e., *P-3* for **5a** and **6a**; *R3* for **7a**). At variance with the structure of the trigold compound **2a**, the orientation of the three P—M fragments is convergent, with all the metal units pointing to the opposite direction of the C—CH_3_ terminus, which also contrasts with the free ligand **1a** (Figure 1), where the phosphine lone pairs are oriented towards the opposite direction. As occurs in the structure of **2a,** there are no metal-metal interactions, either intra- or intermolecularly (all intermetallic distances > 4.5 Å). Bond angles and distances lie within normal values for monovalent phosphinite complexes of these metals. The structures of **5a** and **6a** are basically equal, with P—O bond distances of 1.63 Å and M—P lengths of 2.283(2) and 2.261(2) Å, respectively. The M—P—O are also almost identical, with values of 116.16(14) (**5a**), 115.89(16) (**6a**), and 115.2(3)° (**7a**). As expected, the *iso-*propyl groups of the cymene fragments in **7a** are oriented away from the *C3* rotation axis to reduce steric constraints.

**Figure 5:**
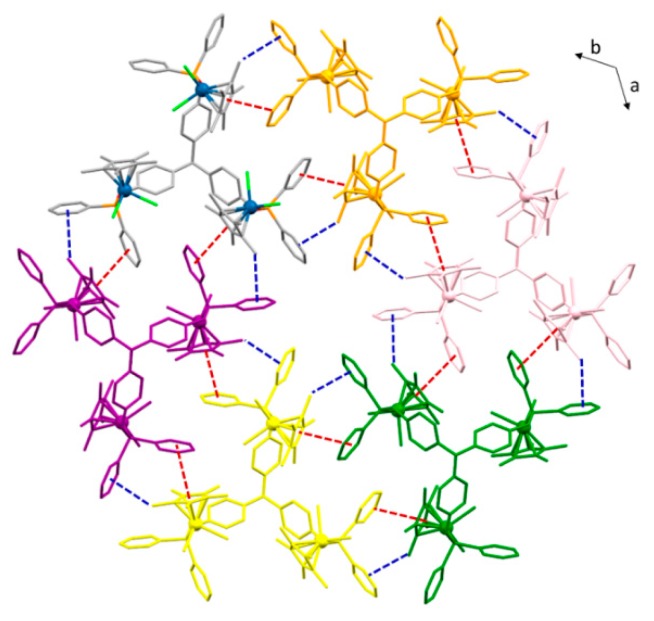
Projection of the crystal structure of **6a** along [0 0 1] highlighting intermolecular π-π (red dotted lines) and C—H/π (blue dotted lines) interactions between triiridium compounds. Each molecule of **6a** has been highlighted in different colors for clarity, while metal centers are represented as balls.

Despite the high similarity of the molecular structures of compounds **5a**, **6a,** and **7a**, the pattern of weak interactions that define their crystal packing differs considerably. For **5a** and **7a,** the trimetallic molecules are not interconnected by direct interactions, instead the presence of considerable amounts of solvent molecules prevents the establishment of an extended covalent network. However, the presence of numerous π-π stacking and C–H/π interactions in the solid-state structure of **6a** gives rise to well-defined *ab* layers of triiridium complexes (Figure 5). An initial analysis shows that each trimetallic motif is connected to three other adjacent ones by two π-π interactions between *η*^5^-C_5_Me_5_ and phenyl phosphinite rings, as well as two weak C—H/π interactions between one sp^3^ CH group of the cyclopentadienyl fragments, and a contiguous phenyl ring. Thus, this pattern gives rise to hexagonal periodic motifs that form the aforementioned *ab* layers. The π-π interactions are characterized by a centroid-centroid distance of 3.772(5) Å, with a plane-plane angle of 11.53(9)° and an offset angle of 6.23(7)° [52]. In turn, the C—H/π contacts are defined by a shortest H···C(sp^2^) distance of 2.900(6) Å; *d*_H···centroid_ of 3.13(1) Å; and C—H···centroid angle of 106.5(5)°, the latter indicating that these interactions are particularly weak [53,54].

**Scheme 4:**
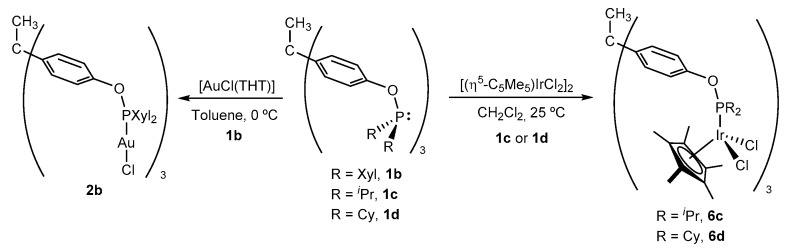
Synthesis of trimetallic complexes based on trisphosphinite ligands **1b**–**d**.

To probe the coordination ability of the reported trisphosphinite ligands **1**, we chose precursor [(*η*^5^-C_5_Me_5_)IrCl_2_]_2_ to prepare the corresponding trimetallic complexes **6b**–**d** (Scheme 4). Following the same experimental procedure that led us to isolate **6a** we could have access to compounds **6c** and **6d** in excellent yields (*ca.* 85%). In contrast, targeting **6b** proved more challenging, and produced a complex mixture of products as evinced by multinuclear NMR analysis, which could be attributed to both steric reasons and to the possibility of phosphine cyclometalation through one or several of the benzylic methyl groups of the xylyl rings, as previously observed in related compounds based on the [(*η*^5^-C_5_Me_5_)Ir] moiety [55,56,57,58]. Nevertheless, we could isolate the corresponding trigold complex [CH_3_C{(C_6_H_4_OP(Xyl)_2_)AuCl}_3_] (**2b**) by reaction between **1b** and [AuCl(THT)], the latter being less sterically congested than the iridium precursor and non-amenable to C(sp^3^)—H cyclometalation. These complexes were characterized by microanalysis and multinuclear NMR spectroscopy, with data in agreement with the previously discussed iridium and gold complexes.

**Figure 6:**
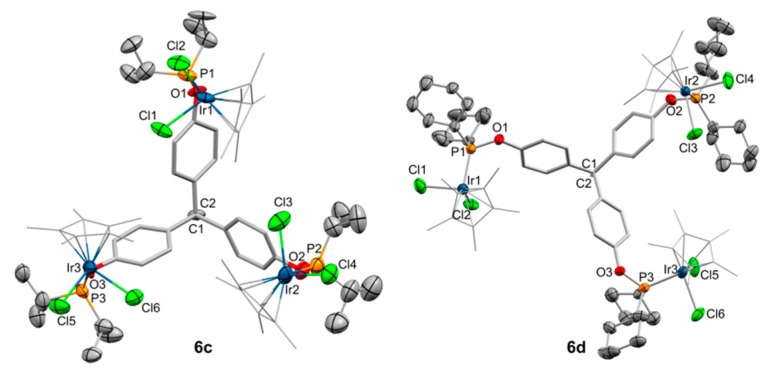
ORTEP view of the solid structure of compounds **6c** and **6d** with thermal ellipsoids set at 50% probability. Hydrogen atoms and solvent molecules have been excluded for the sake of clarity. Selected bond distances (Å) and angles (°): Compound **6c**: Ir1–P1, 2.295(6); Ir2–P2, 2.296(7); Ir3–P3, 2.302(8); Ir1–Cl2. 2.396(6); Ir1–Cl1. 2.420(6); Ir2–Cl3, 2.398(8); Ir2–Cl4, 2.398(8); Ir3–Cl6, 2.398(7); Ir3–Cl5, 2.406(6); P1–O1, 1.637(17); P2–O2, 1.65(2); P3–O3, 1.646(19); C1–C2, 1.62(3); P1–Ir1–Cl2, 92.6(2); P1–Ir–Cl1, 92.1(2); Cl2–Ir1–Cl1, 85.0(2); P2–Ir2–Cl3, 90.0(3); P2–Ir2–Cl4, 88.6(3); P3–Ir3–Cl6, 89.9(3); P3–Ir3–Cl5, 92.1(2). Compound **6d**: Ir1–P1, 2.2801(14); Ir1–Cl1, 2.3940(17); Ir1–Cl2, 2.4003(16); Ir2–P2, 2.3088(14); Ir2–Cl4, 2.3896(17); Ir2–Cl3, 2.4066(16); Ir3–P3, 2.2862(15); Ir3–Cl5, 2.4000(14); Ir3–Cl6, 2.4146(16); P1–O1, 1.642(4); P2–O2, 1.645(4); P3–O3, 1.638(4); C1–C2, 1.562(7); P1–Ir1–Cl1, 92.05(6); C21–Ir1–Cl2, 98.65(18); P1–Ir1–Cl2, 92.07(5); Cl1–Ir1–Cl2, 86.82(7); P2–Ir2–Cl4, 91.22(5); P2–Ir2–Cl3, 87.27(5); P3–Ir3–Cl5, 90.89(5); P3–Ir3–Cl6, 89.09(6); Cl5–Ir3–Cl6, 89.02(7); O1–P1–Ir1, 112.94(15); O2–P2–Ir2, 115.71(14); O3–P3–Ir3, 113.42(17).

Single crystals of good quality were grown for compounds **6c** and **6d** by pentane/chloroform diffusion and toluene evaporation, respectively. Those permitted us to further corroborate their molecular formulation by X-ray diffraction analysis (Figure 6). Bond angles and distances are comparable to those in compound **6a** and do not require further discussion. Interestingly, the structure of **6d**, along with that of **2a**, are the only ones within this work that present alternate configurations of the phosphinite moieties in the solid state. While one of the O—P vectors in **6d** points away from the C—CH_3_ direction (‘torsion angle’ of 135.8°), in the same manner as in the rest of trimetallic structures of this work, the other two O—P bonds are placed in an alternate fashion (‘torsion angles’ of 43.5 and 57.2°). It seems that this peculiar rearrangement does not involve the establishment of new intramolecular weak interactions, but it certainly demonstrates the flexibility of the multidentate ligands reported in this work, even after coordination of three metal fragments.

## 3. Materials and Methods

**Figure 7:**
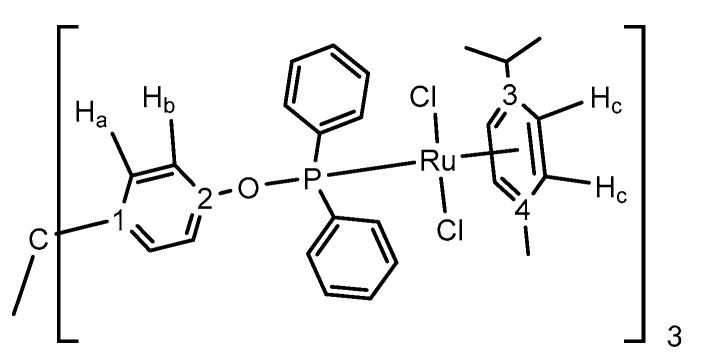
Labeling scheme used for ^1^H and ^13^C{^1^H} NMR assignments.

**General considerations.** All preparations and manipulations were carried out using standard Schlenk and glove-box techniques under an atmosphere of high purity nitrogen. All solvents were dried, stored over 4 Å molecular sieves, and degassed prior to use. Dichloromethane (CH_2_Cl_2_) was distilled under nitrogen over CaH_2_. Toluene (C_7_H_8_) and *n*-pentane (C_5_H_12_) were distilled under nitrogen over sodium. Tetrahydrofuran (THF) and diethyl ether were distilled under nitrogen over sodium/benzophenone. [D_6_] Benzene was dried over sodium, while CDCl_3_ and CD_2_Cl_2_ over molecular sieves (4 Å) and distilled under nitrogen. Et_3_N was dried over KOH and distilled over nitrogen. [AuCl(THT)] (THT = tetrahydrothiophene) [59], [(*η*^5^-C_5_Me_5_)RhCl_2_]_2_ and [(*η*^5^-C_5_Me_5_)IrCl_2_]_2_ [60], [(*η*^6^-C_10_H_14_)RuCl_2_]_2_ [61], and chlorodixylyl phosphine [57] were prepared as described previously. Other chemicals were commercially available and used as received. Solution NMR spectra were recorded on Bruker AMX-300, DRX-400, and DRX-500 spectrometers. Spectra were referenced to external SiMe_4_ (δ: 0 ppm) using the residual proton solvent peaks as internal standards (^1^H NMR experiments), or the characteristic resonances of the solvent nuclei (^13^C NMR experiments), while ^31^P was referenced to H_3_PO_4_. Spectral assignments were made by routine one- and two-dimensional NMR experiments where appropriate (Figure 7). For elemental analyses, a LECO TruSpec CHN elementary analyzer was utilized. CCDC 1973685-1973691 contain the supplementary crystallographic data for this paper. These data can be obtained free of charge from The Cambridge Crystallographic Data Centre.

**Synthesis of trisphosphinites. Method A (ligands 1a and 1c).** To a Et_2_O (5 mL) solution of 1,1,1-tris(4-hydroxyphenyl)-ethane (80.0 mg, 0.26 mmol) was added Et_3_N (365 µL, 2.63 mmol) at room temperature and under nitrogen atmosphere. Then, the corresponding chlorophosphine (PClPh_2_: 170 µL, 0.92 mmol; PCl*^i^*Pr_2_: 145 µL, 0.92 mmol) was added dropwise. The solution was stirred at room temperature overnight, after which it was filtrated and concentrated (to ca. 1 mL) under vacuum. Compound **1a** was isolated as a white solid (189 mg, 0.22 mmol, 84%) by precipitation with pentane (5–10 mL) and after filtering off and drying the solid under reduced pressure. Compound **1c** was isolated as a colorless oil (109 mg, 0.17 mmol, 64%) after evaporation of the Et_2_O solution and used without further purification. **Method B**. To a THF (5 mL) suspension of 1,1,1-tris(4-hydroxyphenyl)-ethane (80.0 mg, 0.26 mmol) and NaH (63.0 mg, 2.63 mmol) in a J. Young ampoule at room temperature, a THF solution of the corresponding phosphine (PCl(Xyl)_2_: 255 mg, 0.92 mmol; PClCy_2_: 215 mg, 0.92 mmol) was added dropwise. The solution was stirred at room temperature for three days. For better yields, Et_3_N (220 µL, 1.58 mmol) was subsequently added to the reaction mixture, and the suspension further stirred overnight. The reaction mixture was filtrated and the solvents were evaporated under vacuum, yielding a colorless oil that was extracted with pentane. The resulting white sticky foam was used without further purification (**1d**: 148 mg, 0.16 mmol, 63%). Crystals of **1b** suitable for X-ray diffraction studies were grown by slow evaporation of the reaction crude (13.2 mg, 0.01 mmol 5%). These compounds were characterized by spectroscopic methods, except for **1c** that also includes a single crystal X-ray diffraction analysis, and **1a** for which elemental analysis is also provided. **Compound 1a.** Anal. Calc. for C_56_H_45_O_3_P_3_**:** C, 78.31; H, 5.28. Found: C, 78.33; H, 5.57. ^1^H NMR (400 MHz, CDCl_3_, 25 °C) δ: 7.60–7.53 (m, 12 H, *o*-C_6_H_5_), 7.42–7.35 (m, 18 H, *m-*C_6_H_5_; *p-*C_6_H_5_), 6.98 (dd, 6 H, ^3^*J*_HH_ = 8.8 Hz, ^4^*J*_H P_ = 1.2 Hz, H_b_), 6.94 (d, 6 H, ^3^*J*_HH_ = 8.8 Hz, H_a_), 2.05 ppm (s, 3 H, CCH_3_). ^13^C {^1^H} NMR (100 MHz, CDCl_3_, 25 °C) δ: 155.5 (d, ^2^*J*_CP_ = 10 Hz, C_2_), 143.5 (s, C_1_), 141.1 (d, ^1^*J*_CP_ = 18 Hz, *ipso*-C_6_H_5_), 130.7 (d, ^2^*J*_CP_ = 22 Hz, *o*-C_6_H_5_), 129.8 (s, CH_a_; *p*-C_6_H_5_), 128.6 (d, ^3^*J*_CP_ = 7 Hz, *m*-C_6_H_5_), 118.1 (d, ^3^*J*_CP_ = 11 Hz, CH_b_), 51.1 (s, *C*CH_3_), 30.5 ppm (s, C*C*H_3_). ^31^P {^1^H} NMR (162 MHz, CDCl_3_, 25 °C) δ: 110.5 ppm. **Compound 1b.** ^1^H NMR (400 MHz, CDCl_3_, 25 °C) δ: 7.12 (t, 6 H, ^3^*J*_HH_ = 7.5 Hz, *p*-C_6_H_5_), 6.99–6.93 (m, 18 H, *m-*C_6_H_5_; H_b_), 6.91 (d, 6 H, ^3^*J*_HH_ = 8.9 Hz, H_a_), 2.37 (s, 36 H, CH_3_ (Xyl)), 2.05 (s, 3 H, CCH_3_). ^13^C {^1^H} NMR (100 MHz, CDCl_3_, 25 °C) δ: 155.1 (d, ^2^*J*_CP_ = 14 Hz, C_2_), 143.0 (s, C_1_), 141.6 (d, ^2^*J*_CP_ = 17 Hz, *o*-Xyl), 136.9 (d, ^1^*J*_CP_ = 27 Hz, *ipso*-Xyl), 129.8 (s, CH_a_), 129.3 (d, ^3^*J*_CP_ = 7 Hz, *m*-Xyl), 129.3 (s, *p*-Xyl), 117.5 (d, ^3^*J*_CP_ = 13 Hz, CH_b_), 51.0 (s, *C*CH_3_), 32.3 (s, C*C*H_3_), 22.5 (d, ^3^*J*_CP_ = 14 Hz, *C*H_3_ (Xyl)). ^31^P {^1^H} NMR (121 MHz, CDCl_3_, 25 °C) δ: 113.5 ppm. **Compound 1c**. ^1^H NMR (300 MHz, CDCl_3_, 25 °C) δ: 6.94 (m, 12 H, H_a_; H_b_), 2.07 (s, 3 H, CCH_3_), 1.91 (septd, 6 H, ^3^*J*_HH_ = 7.0 Hz, ^2^*J*_HP_ = 2.2 Hz, CH (^i^Pr)), 1.17 (dd, 18 H, ^3^*J*_HH_ = 7.0 Hz, ^3^*J*_HP_ = 10.7 Hz, CH_3_ (^i^Pr)), 1.09 ppm (dd, 18 H, ^3^*J*_HH_ = 7.2 Hz, ^3^*J*_HP_ = 15.9 Hz, CH_3_ (^i^Pr)). ^13^C {^1^H} NMR (75 MHz, CDCl_3_, 25 °C) δ: 157.6 (d, ^2^*J*_CP_ = 9 Hz, C_2_), 143.1 (s, C_1_), 129.9 (s, CH_a_) 118.1 (d, ^3^*J*_CP_ = 10 Hz, CH_b_), 51.1 (s, *C*CH_3_), 31.2 (s, C*C*H_3_), 28.7 (d, ^1^*J*_CP_ = 17 Hz, *C*H (^i^Pr)), 18.2 (d, ^2^*J*_CP_ = 20 Hz, *C*H_3_ (^i^Pr)), 17.4 ppm (d, ^2^*J*_CP_ = 8 Hz, *C*H_3_ (^i^Pr)). ^31^P {^1^H} NMR (121 MHz, CDCl_3_, 25 °C) δ: 149.4 ppm. **Compound 1d**. ^1^H NMR (400 MHz, CD_2_Cl_2_, 25 °C) δ: 7.00 (m, 12 H, H_a_; H_b_), 2.11 (s, 3 H, CCH_3_), 2.00–1.27 ppm (m, 66 H, CH (Cy); CH_2_ (Cy)). ^13^C {^1^H} NMR (100 MHz, CD_2_Cl_2_, 25 °C) δ: 158.4 (d, ^2^*J*_CP_ = 9 Hz, C_2_), 143.4 (s, C_1_), 130.2 (s, CH_a_), 118.5 (d, ^3^*J*_CP_ = 10 Hz, CH_b_), 51.6 (s, *C*CH_3_), 38.9 (d, ^1^*J*_CP_ = 18 Hz, CH_2_ (Cy)), 31.5 (s, C*C*H_3_), 28.9 (d, ^2^*J*_CP_ = 19 Hz, CH_2_ (Cy)), 27.8 (s, CH_2_ (Cy)), 27.7 (d, ^3^*J*_CP_ = 6 Hz, CH_2_ (Cy)), 27.6 (s, CH_2_ (Cy)), 27.3 ppm (s, CH_2_ (Cy)). ^31^P {^1^H} NMR (162 MHz, CDCl_3_, 25 °C) δ: 144.6 ppm.

**Compound 2a.** A dichloromethane (5 mL) solution of **1a** (334 mg, 0.39 mmol) was slowly added at 0 °C and under nitrogen atmosphere over a dichloromethane (5 mL) solution of [AuCl(THT)] (374 mg, 1.17 mmol). The solution was stirred at 0 °C for 5 h, then the solvent was concentrated under vacuum, and pentane addition caused the precipitation of a white solid, which was further washed with pentane for several times yielding compound **2a** as a white solid (580 mg, 0.37 mmol, 95%). Single crystals were obtained by slow diffusion of pentane into a benzene solution (3:1). Anal. Calc. for C_56_H_45_Cl_3_O_3_P_3_Au_3_: C, 43.22; H, 2.91. Found: C, 43.45; H, 3.25. ^1^H NMR (400 MHz, CD_2_Cl_2_, 25 °C) δ: 7.92–7.82 (m, 12 H, *o*-C_6_H_5_), 7.67–753 (m, 18 H, *m-* C_6_H_5_; *p-*C_6_H_5_), 7.05 (dd, 6 H, ^3^*J*_HH_ = 8.9 Hz, ^4^*J*_HP_ = 1.2 Hz, H_b_), 6.96 (d, 6 H, ^3^*J*_HH_ = 8.9 Hz, H_b_), 2.12 ppm (s, 3 H, CCH_3_). ^13^C{^1^H} NMR (100 MHz, CD_2_Cl_2_, 25 °C) δ: 153.2 (d, ^2^*J*_CP_ = 5 Hz, C_2_), 146.3 (s, C_1_), 133.8 (s, *p*-C_6_H_5_), 133.3 (d, ^1^*J*_CP_ = 70 Hz, *ipso*-C_6_H_5_), 133.0 (d, ^2^*J*_CP_ = 16 Hz, *o*-C_6_H_5_), 131.0 (s, CH_a_), 130.0 (d, ^3^*J*_CP_ = 12 Hz, *m*-C_6_H_5_), 120.5 (d, ^3^*J*_CP_ = 7 Hz, CH_b_), 52.2 (s, *C*CH_3_), 30.9 ppm (s, C*C*H_3_). ^31^P{^1^H} NMR (162 MHz, CD_2_Cl_2_, 25 °C) δ: 112.8 ppm.

**Compound 2b.** To a toluene solution (1–2 mL) of [AuTHTCl] (12.4 mg, 0.04 mmol) in a Schlenk flask was added dropwise a toluene solution (1–2 mL) of **1b** (13.3 mg, 0.01 mmol) at 0 °C. The resulting solution was stirred for 1 h, then the solvent was concentrated up to the minimum volume and precipitated with pentane, washed with pentane (2 × 3 mL) yielding a pure white solid (7.000 mg, 31% yield). Anal. Calc. for C_68_H_69_Cl_3_O_3_P_3_Au_3_: C, 47.36; H, 4.03. Found: C, 46.94; H, 3.98. ^1^H NMR (400 MHz, CD_2_Cl_2_, 25 °C) δ: 7.34 (t, 6 H, ^3^*J*_HH_ = 7.5 Hz, *p*-C_6_H_5_), 7.17–7.05 (m, 12 H, *m-*C_6_H_5_), 6.95 (d, 6 H, ^3^*J*_HH_ = 8.7 Hz, H_b_), 6.90 (d, 6 H, ^3^*J*_HH_ = 8.7 Hz, H_a_), 2.56 (s, 36 H, CH_3_ (Xyl)), 2.07 ppm (s, 3 H, CCH_3_). ^13^C{^1^H} NMR (100 MHz, CD_2_Cl_2_, 25 °C) δ: 152.9 (d, ^2^*J*_CP_ = 5 Hz, C_2_), 146.0 (s, C_1_), 142.3 (d, ^2^*J*_CP_ = 12 Hz, *o*-Xyl), 132.9 (s, *p*-Xyl), 131.7 (d, ^3^*J*_CP_ = 9 Hz, *m*-Xyl), 131.0 (d, ^1^*J*_CP_ = 64 Hz, *ipso*-Xyl), 130.9 (s, CH_a_), 120.2 (d, ^3^*J*_CP_ = 7 Hz, CH_b_), 52.1 (s, *C*CH_3_), 30.9 (s, C*C*H_3_), 24.5 ppm (d, ^3^*J*_CP_ = 9 Hz, *C*H_3_ (Xyl)). ^31^P{^1^H} NMR (202 MHz, CD_2_Cl_2_, 25 °C) δ: 103.6 ppm.

**Compound 3a.** To a CH_2_Cl_2_ solution (~2–3 mL) of [AgOTf] (OTf^−^ = OSO_2_CF_3_^−^) (43.4 mg, 0.17 mmol) in a Schlenk flask was slowly added a CH_2_Cl_2_ solution (~2–3 mL) of **1** (50.0 mg, 0.06 mmol) at room temperature in the absence of light. The resulting solution was stirred for 3–4 h. After this, the solvent was concentrated up to the minimum volume and precipitated with pentane, washed with pentane (2 × 5mL) and diethyl ether (1 × 3 mL) yielding a pure white solid (68.0 mg, 0.04 mmol, 72% yield) that remain stable under air for several hours. Anal. Calc. for C_59_H_45_F_9_O_12_P_3_S_3_Ag_3_: C,43.48; H, 2.78; S, 5.90. Found: C, 43.28; H, 3.12; S, 5.70. ^1^H NMR (400 MHz, CD_2_Cl_2_, 25 °C) δ: 7.80–7.68 (m, 12 H, *o*-C_6_H_5_), 7.60–7.45 (m, 18 H, *m-*C_6_H_5_; *p-*C_6_H_5_), 7.03 (d, 6 H, ^3^*J*_HH_ = 8.6 Hz, H_a_), 6.97 (d, 6 H, ^3^*J*_HH_ = 8.3 Hz, H_b_), 2.05 ppm (s, 3 H, CCH_3_). ^13^C{^1^H} NMR (100 MHz, CD_2_Cl_2_, 25 °C) δ: 155.1 (s, C_2_), 146.3 (s, C_1_), 134.4 (d, ^1^*J*_CP_ = 38 Hz, *ipso*-C_6_H_5_), 133.2 (s, *p*-C_6_H_5_), 132.3 (d, ^2^*J*_CP_ = 19 Hz, *o*-C_6_H_5_), 131.2 (s, CH_a_), 130.0 (d, ^3^*J*_CP_ = 11 Hz, *m*-C_6_H_5_), 119.9 (d, ^3^*J*_CP_ = 9 Hz, CH_b_), 52.1 (s, *C*CH_3_), 30.7 ppm (s, C*C*H_3_). ^19^F{^1^H} NMR (376 MHz, CD_2_Cl_2_, 25 °C) δ: −77.4 ppm. ^31^P{^1^H} NMR (202 MHz, CD_2_Cl_2_, 25 °C) δ: 120.6 ppm.

**Compound 4a.** To a CH_2_Cl_2_ solution (7–8 mL) of CuCl (30.0 mg, 0.30 mmol) in a Schlenk flask was added dropwise a CH_2_Cl_2_ solution (3–4 mL) of **1** (89.1 mg, 0.10 mmol) at room temperature. The resulting solution was left stirred overnight (~14 h). After this, upon concentration, the solution turns into a white residue that was washed with pentane, diethyl ether and toluene, yielding a pure white solid (97.1 mg, 0.08 mmol, 81% yield) that remain stable under air for several hours. Anal. Calc. for C_56_H_45_Cl_3_O_3_P_3_Cu_3_: C, 58.19; H, 3.92. Found: C, 57.86; H, 4.14. ^1^H NMR (400 MHz, CDCl_3_, 25 °C) δ: 7.77–7.55 (m, 12 H, *o*-C_6_H_5_), 7.37–7.18 (m, 18 H, *m-*C_6_H_5_; *p-*C_6_H_5_), 6.71–6.56 (m, 6 H, H_b_), 6.56–6.38 (m, 6 H, H_a_), 1.76 ppm (s, 3 H, CCH_3_). ^13^C{^1^H} NMR (100 MHz, CDCl_3_, 25 °C) δ: 153.0 (s, C_2_), 143.8 (s, C_1_), 131.4 (s, ^2^*J*_CP_ = 9 Hz, *o*-C_6_H_5_), 130.9 (s, *p*-C_6_H_5_), 129.8 (s, CH_a_), 128.8 (s, ^1^*J*_CP_ = 81 Hz, *ipso*-C_6_H_5_), 128.7 (s, *m*-C_6_H_5_), 118.6 (s, CH_b_), 50.8 (s, *C*CH_3_), 30.5 ppm (s, C*C*H_3_). ^31^P{^1^H} NMR (162 MHz, CDCl_3_, 25 °C) δ: 96.1 ppm (br s).

**General synthesis of group 9 compounds.** A dichloromethane (5 mL) solution of a trisphosphinite ligand **1a** (92.7 mg, 0.11 mmol; 68.7 mg, 0.08 mmol), **1c** (81.5 mg, 0.12 mmol), or **1d** (63.5 mg, 0.07 mmol) were added at room temperature and under nitrogen atmosphere over a dichloromethane (5 mL) solution of the corresponding transition metal precursor ([(*η*^5^-C_5_Me_5_)RhCl_2_]_2_: 164.0 mg, 0.092 mmol for **5a**; [(*η*^5^-C_5_Me_5_)IrCl_2_]_2_: 92.1 mg, 0.23 mmol for **6a**; 139mg, 0.17 mmol for **6c**; 84.8 mg, 0.11 mmol for **6d**). The resulting solution was stirred for one hour, and then the solvent was concentrated at reduced pressure. A red solid for **5a** or a yellow-orange for **6a** were precipitated after addition of pentane, and it was subsequently washed with pentane or diethyl ether for several times yielding the corresponding trimetallic compounds (**5a**: 164 mg, 0.09 mmol 84%; **6a**: 130 mg, 0.06 mmol 83%; **6c**: 192 mg, 0.10 mmol, 84%; **6d**: 130 mg, 0.06 mmol, 87%). Single crystals were obtained by either slow diffusion of diethyl ether into a dichloromethane solution (3:1) of the compound at 25 °C (**5a**, **6a**, **6c**) or by slow evaporation of a toluene solution of the compound (**6d**). **Compound 5a.** Anal. Calc. for C_86_H_90_Cl_6_O_3_P_3_Rh_3_: C, 57.84; H, 5.08. Found: C, 57.93; H, 5.46. ^1^H NMR (400 MHz, CD_2_Cl_2_, 25 °C) δ: 8.15 (m, 12 H, *o*-C_6_H_5_), 7.42–7.32 (m, 18 H, *m-*C_6_H_5_; *p-*C_6_H_5_), 7.28 (d, 6 H, ^3^*J*_HH_ = 8.7 Hz, H_b_), 6.75 (d, 6 H, ^3^*J*_HH_ = 8.8 Hz, H_a_), 1.97 (s, 3 H, CCH_3_), 1.31 ppm (d, 45 H, ^4^*J*_HP_ = 3.7 Hz, C_5_Me_5_). ^13^C{^1^H} NMR (100 MHz, CD_2_Cl_2_, 25 °C) δ: 151.2 (d, ^2^*J*_CP_ = 6 Hz, C_2_), 144.8 (s, C_1_), 136.0 (d, ^1^*J*_CP_ = 47 Hz, *ipso*-C_6_H_5_), 133.3 (d, ^2^*J*_CP_ = 12 Hz, *o*-C_6_H_5_), 131.9 (s, *p*-C_6_H_5_), 130.3 (s, CH_a_), 128.7 (d, ^3^*J*_CP_ = 10 Hz, *m*-C_6_H_5_), 120.4 (d, ^3^*J*_CP_ = 6 Hz, CH_b_), 101.8 (d, ^2^*J*_CP_ = 4 Hz, *C*_5_Me_5_), 51.6 (s, *C*CH_3_), 30.9 (s, C*C*H_3_), 9.4 ppm (s, C_5_*Me*_5_). ^31^P{^1^H} NMR (121 MHz, CD_2_Cl_2_, 25 °C) δ: 113.7 ppm (d, ^1^*J*_PRh_ = 170 Hz). **Compound 6a.** Anal. Calc. for C_86_H_90_Cl_6_O_3_P_3_Ir_3_: C, 50.29; H, 4.42. Found: C, 50.23; H, 4.66. MS (ESI) *m/z* Calc. for [M + H − Cl]^+^:_2041.3. Expt.: 2041.1. ^1^H NMR (400 MHz, CD_2_Cl_2_, 25 °C) δ: 8.21–8.10 (m, 12 H, *o*-C_6_H_5_), 7.43-7.312 (m, 18 H, *m-*C_6_H_5_; *p-*C_6_H_5_), 7.25 (d, 6 H, ^3^*J*_HH_ = 8.8 Hz, H_b_), 6.79 (d, 6 H, ^3^*J*_HH_ = 8.9 Hz, H_a_), 1.99 (s, 3 H, CCH_3_), 1.30 ppm (d, 45 H, ^4^*J*_HP_ = 2.2 Hz, C_5_Me_5_). ^13^C{^1^H} NMR (100 MHz, CD_2_Cl_2_, 25 °C) δ: 151.0 (d, ^2^*J*_CP_ = 6 Hz, C_2_), 144.6 (s, C_1_), 137.4 (d, ^1^*J*_CP_ = 60 Hz, *ipso*-C_6_H_5_), 133.0 (d, ^2^*J*_CP_ = 12 Hz, *o*-C_6_H_5_), 131.7 (s, *p*-C_6_H_5_), 130.3 (s, CH_a_), 128.6 (d, ^3^*J*_CP_ = 11 Hz, *m*-C_6_H_5_), 120.0 (d, ^3^*J*_CP_ = 6 Hz, CH_b_), 95.9 (s, *C*_5_Me_5_), 51.6 (s, *C*CH_3_), 31.0 (s, C*C*H_3_), 8.9 ppm (s, C_5_*Me*_5_). ^31^P{^1^H} NMR (121 MHz, CD_2_Cl_2_, 25 °C) δ: 72.3. ppm. **Compound 6c**. Anal. Calc. for C_68_H_102_Cl_6_O_3_P_3_Ir_3_: C, 44.15; H, 5.56. Found: C, 44.39; H, 5.61. ^1^H NMR (400 MHz, CD_2_Cl_2_, 25 °C) δ: 7.26 (d, 6 H, ^3^*J*_HH_ = 8.8 Hz, H_b_), 6.98 (d, 6 H, ^3^*J*_HH_ = 8.6 Hz, H_a_), 3.36–3.21 (m, 6 H, CH (^i^Pr)), 2.12 (s, 3 H, CCH_3_), 1.43 (overlapped s, 45 H, C_5_Me_5_), 1.42 (overlapped dd, 18 H, ^3^*J*_HH_ = 7.5 Hz, ^3^*J*_HP_ = 16.4 Hz, CH_3_ (^i^Pr)), 1.28 (dd, 18 H, ^3^*J*_HH_ = 7.3 Hz, ^3^*J*_HP_ = 13.9 Hz, CH_3_ (^i^Pr)). ^13^C{^1^H} NMR (100 MHz, CD_2_Cl_2_, 25 °C) δ: 152.1 (d, ^2^*J*_CP_ = 7 Hz, C_2_), 144.2 (s, C_1_), 130.6 (s, CH_a_), 119.2 (d, ^3^*J*_CP_ = 5 Hz, CH_b_), 94.8 (s, *C*_5_Me_5_), 51.8 (s, *C*CH_3_), 32.6 (d, ^1^*J*_CP_ = 30 Hz, CH (^i^Pr)), 31.4 (s, C*C*H_3_), 20.0 (d, ^2^*J*_CP_ = 4 Hz, CH_3_ (^i^Pr)), 18.2 (s, CH_3_ (^i^Pr)), 9.7 (s, C_5_*Me*_5_).^31^P{^1^H} NMR (162 MHz, CD_2_Cl_2_, 25 °C) δ: 108.5 ppm. **Compound 6d.** Anal. Calc. for C_86_H_126_Cl_6_O_3_P_3_Ir_3_: C, 49.42; H, 6.08. Found: C, 49.40; H, 5.99. ^1^H NMR (400 MHz, CDCl_3_, 25 °C) δ: 7.25 (d, 6 H, ^3^*J*_HH_ = 9.1 Hz, H_b_), 6.94 (d, 6 H, ^3^*J*_HH_ = 8.6 Hz, H_a_), 3.11–2.92 (m, 6 H, CH (Cy)), 2.78–2.62 (m, 6 H, CH_2_ (Cy)), 2.11–1.97 (overlapped m, 6 H, CH_2_ (Cy)), 2.07 (overlapped s, 3 H, CH_3_), 1.89–1.06 (overlapped m, 46 H, CH_2_ (Cy)), 1.45 ppm (overlapped s, 45 H, C_5_Me_5_). ^13^C{^1^H} NMR (100 MHz, CDCl_3_, 25 °C) δ: 151.5 (d, ^2^*J*_CP_ = 7 Hz, C_2_), 143.4 (s, C_1_), 129.9 (s, CH_a_), 118.5 (d, ^3^*J*_CP_ = 4 Hz, CH_b_), 94.0 (s, *C*_5_Me_5_), 51.1 (s, *C*CH_3_), 43.3 (d, ^1^*J*_CP_ = 29 Hz, CH (Cy)), 30.9 (s, C*C*H_3_), 29.3 (d, ^3^*J*_CP_ = 3 Hz), 27.9 (d, ^2^*J*_CP_ = 10 Hz, CH_2_ (Cy)), 27.6 (s, CH_2_ (Cy)), 27.5 (d, ^2^*J*_CP_ = 14 Hz, CH_2_ (Cy)), 26.7 (s, CH_2_ (Cy)), 9.2 ppm (s, C_5_*Me*_5_). ^31^P{^1^H} NMR (162 MHz, CDCl_3_, 25 °C) δ: 103.2 ppm.

**Compound 7a.** A dichloromethane (5 mL) solution of **1a** (66.0 mg, 0.08 mmol) was added at room temperature and under nitrogen atmosphere over a dichloromethane (5 mL) solution of [Ru(*p*-cymene)Cl_2_]_2_ (70.7 mg, 0.23 mmol). The resulting solution was stirred for 1 h. Then the solvent was concentrated under, vacuum pentane was added to afford the precipitation of a red solid, which was washed with pentane and diethyl ether. The resulting solid was passed through a pad of SiO_2_ in ethyl acetate to obtain **7a** as a red powder (93.2 mg, 0.05 mmol, 68%). Single crystals were obtained by slow diffusion of pentane into a CH_2_Cl_2_/acetone solution (3:1) at −30 °C. Anal. Calc. for C_86_H_87_Cl_6_O_3_P_3_Ru_3_: C, 58.11; H, 4.93. Found: C, 57.97; H, 4.92. ^1^H NMR (400 MHz, CDCl_3_, 25 °C) δ: 8.05–7.92 (m, 12 H, *o*-C_6_H_5_), 7.39–7.28 (m, 18 H, *m-*C_6_H_5_; *p-*C_6_H_5_), 7.08 (d, 6 H, ^3^*J*_HH_ = 8.5 Hz, H_b_), 6.74 (d, 6 H, ^3^*J*_HH_ = 8.4 Hz, H_a_), 5.29–5.22 (m, 12 H, H_c_), 2.51 (sept, 3 H, ^3^*J*_HH_ = 7.3 Hz, CH (^i^Pr) (*p*-cymene)), 1.92 (s, 3 H, CCH_3_), 1.46 (s, 9 H, CH_3_ (*p*-cymene)), 0.83 ppm (d, 18 H, ^3^*J*_HH_ = 6.9 Hz, CH_3_ (^i^Pr) (*p*-cymene)). ^13^C{^1^H} NMR (100 MHz, CDCl_3_, 25 °C) δ: 151.2 (s, C_2_), 144.2 (s, C_1_), 136.3 (d, ^1^*J*_CP_ = 48 Hz, *ipso*-C_6_H_5_), 132.4 (d, ^2^*J*_CP_ = 10 Hz, *o*-C_6_H_5_), 131.2 (s, *p*-C_6_H_5_), 129.8 (s, CH_a_), 128.2 (d, ^3^*J*_CP_ = 10 Hz, *m*-C_6_H_5_), 120.3 (s, CH_b_), 110.4 (s, C_3_), 97.2 (s, C_4_), 92.2 (s, CH_c_), 87.6 (d, ^2^*J*_CP_ = 5 Hz, CH_c_), 51.2 (s, *C*CH_3_), 31.0 (s, C*C*H_3_), 30.4 (s, CH (^i^Pr) (*p*-cymene)), 21.8 (s, CH_3_ (^i^Pr) (*p*-cymene)), 17.6 ppm (s, CH_3_ (*p*-cymene)). ^31^P{^1^H} NMR (121 MHz, CDCl_3_, 25 °C) δ: 113.8 ppm.

## 4. Conclusions

In summary, we have described the coordination chemistry of a new family of readily accessible trisphosphinite ligands by preparing a number of trimetallic complexes based on a range of late transition metal precursors (i.e., Au(I), Ag(I), Cu(I), Ir(III), Rh(III), and Ru(II)). These ligands present a remarkable degree of conformational flexibility, as evinced by the X-ray structures of one of the free ligands and several of the trimetallic complexes. This contrasts with trisphosphines typically employed to construct supramolecular architectures, which rely on rigid backbones. Thus, trisphosphinites could offer unique features to design novel self-assembled structures and complement the still underdeveloped area of multidentate phosphines in the context of supramolecular chemistry. This work offers key information on the coordination ability of these systems that will be valuable for future work in that direction. 

## Supplementary Materials

The following are available online, Figure S1–S40: NMR spectra of new compounds, Figure S41: MS (ESI) of **6a**, Table S1–S2: X-Ray Structural Data of new compounds.
