# Supplementary material for: A Versatile Approach to Access Trimetallic Complexes Based on Trisphosphinite Ligands
Source: Molecules. 2020 Jan 29;25(3):593. doi: 10.3390/molecules25030593 (PMC7037439; doi:10.3390/molecules25030593)
Supplement: Supplementary file 1 [file molecules-25-00593-s001.pdf]

A versatile approach to access trimetallic complexes based on trisphosphinites ligands

Juan Miranda-Pizarro [1], Macarena G. Alférez [1], M. Dolores Fernández-Martínez [1], Eleuterio Álvarez [1], Celia Maya [1] and Jesús Campos [1,*]

[1]Instituto de Investigaciones Químicas (IIQ), Departamento de Química Inorgánica and Centro de Innovación en Química Avanzada (ORFEO-CINQA). Universidad de Sevilla and Consejo Superior de Investigaciones Científicas (CSIC). Avenida Américo Vespucio 49, 41092 Sevilla (Spain).

*jesus.campos@iiq.csic.es

SUPPORTING INFORMATION

1. X-Ray Structural Characterization of new compounds S2

2. NMR spectra of new compounds S5

3. MS (ESI) spectrum of compound **6a** S25

4. References S26

1. X-Ray Structural Characterization of new compounds

Single crystals of suitable size of each compound were selected and covered with FOMBLIN oil and mounted on a glass fiber. Data collections have been performed either on a Bruker-AXSX8Kappa diffractometer equipped with an Apex-II CCD area detector, using a graphite monochromator Ag Kα1 (λ=0.56086 Å) and a Bruker Cryo-Flex low-temperature device (structure **6a**; Centro de Investigación, Tecnología e Innovación, Sevilla University) or on a Bruker APEX-II CCD diffractometer using monochromatic radiation λ(Mo $K_{\alpha 1}$) = 0.71073 Å. (structures **1b**, **2a**, **5**, **6c**, **6d**, **7a**; Instituto de Investigaciones Químicas, Sevilla). In both cases data collections were processed with APEX-W2D-NT (Bruker, 2004), cell refinement and data reduction with SAINT-Plus (Bruker, 2004) and the absorption was corrected by multiscan method applied by SADABS.[1] The space-group assignment was based upon systematic absences, E statistics, and successful refinement of the structure. The structure was solved by direct methods and expanded through successive difference Fourier maps, F^2 (SHELXTL).[2] In the last cycles of refinement, ordered non-hydrogen atoms were refined anisotropically. Hydrogen atoms connected to carbon atoms were included in idealized positions, and a riding model was used for their refinement. In all structures except in **6a** refinement showed residual electron density due to heavily disordered solvent molecules which could not be modelled. Therefore the option SQUEEZE of the program package PLATON[3] was used to create an hkl file taking into account the residual electron density in the void areas.

A summary of all crystallographic data and refinement parameters for each compound is provided in Table SX. Atomic coordinates, anisotropic displacement parameters and bond lengths and angles can be found in the cif files which have been deposited in the Cambridge Crystallographic Data Centre with no. 1973685-1973691. These data can be obtained free of charge from The Cambridge Crystallographic Data Centre via www.ccdc.cam.ac.uk/data_request/cif.

Table S1. Crystal data and structure refinement for compounds **1b**, **2a**, **5a** and **6a**.

	**1b**	**2a**	**5a**	**6a**
formula	$C_{68}H_{69}O_3P_3$	$C_{59}H_{48}Au_3Cl_3O_3P_3$	$C_{86}H_{90}Cl_6O_3P_3Rh_3$	$C_{86}H_{90}Cl_6Ir_3O_3P_3$ [+ solvent]
fw	1027.14	1595.13	1785.91	513.45
cryst.size, mm	0.40 x 0.25 x 0.20	0.35 x 0.21 x 0.17	0.23 x 0.19 x 0.13	0.29 x 0.19 x 0.14
crystal system	Monoclinic	Monoclinic	Trigonal	Trigonal
space group	C2/c	$P2_1/c$	P -3	P -3
a, Å	37.624(4)	9.2801(5)	23.638(3)	23.4482(8)
b, Å	7.3677(8)	16.5576(8)	23.638(3)	23.4482(8)
c, Å	42.797(4)	38.7124(16)	11.5473(15)	11.3520(7)
α, deg	90	90	90	90
β, deg	102.413(6)	96.484(2)	90	90
γ, deg	90	90	120	120
V, Å^3	11586(2)	5910.3(5)	5587.5(15)	5405.3(5)
T, K	193(2)	196(2)	196(2)	173(2)
Z	8	4	9	2
ρ_{calc}, g cm^{-3}	1.178	1.793	1.062	1.262
μ, mm^{-1} (MoKα)	0.149	7.686	0.66	2.124
F(000)	4368	3036	1824	2016
absorption corrections	multi-scan, 0.66-0.75	multi-scan, 0.59-0.75	multi-scan, 0.59-0.75	multi-scan, 0.64-0.75
θ range, deg	1.11 – 25.25	1.22 – 25.25	1.76 – 30.63	1.37 – 22.24
no. of rflns measd	68106	47059	61290	50526
R_{int}	0.050	0.068	0.094	0.038
no. of rflns unique	10464	11015	61290	9062
no. of params / restraints	680 / 988	641 /18	311 / 0	310 / 0
R_1 ($I > 2\sigma(I)$) [a]	0.055	0.051	0.083	0.053
R_1 (all data)	0.071	0.093	0.140	0.060
wR_2 ($I > 2\sigma(I)$)	0.200	0.127	0.212	0.176
wR_2 (all data)	0.218	0.157	0.228	0.179
Diff.Fourier.peaks min/max, eÅ^{-3}	-0.541 / 0.544	-1.583 / 2.696	-1.134 / 1.088	-5.494 / 1.687
CCDC number	1973688	1973690	1973689	1973687

Table S2. Crystal data and structure refinement for compounds **6c**, **6d** and **7a**.

	**6c**	**6d**	**7a**
formula	$C_{139} H_{207} Cl_{21} Ir_6 O_6 P_6$	$C_{93} H_{134} Cl_6 Ir_3 O_3 P_3$	$C_{86} H_{87} Cl_6 O_3 P_3 Ru_3$ [+ solvent]
fw	4057.50	2182.20	1184.91
cryst.size, mm	0.25 x 0.20 x 0.15	0.50 x 0.30 x 0.10	0.29 x 0.21 x 0.15
crystal system	Monoclinic	Triclinic	Trigonal
space group	$P2_1$	P 1	R3
a, Å	21.0882(3)	11.7488(14)	20.5919(9)
b, Å	27.3740(4)	14.934(2)	20.5919(9)
c, Å	35.1998(6)	27.216(4)	48.037(3)
α, deg	90	99.694(5)	90
β, deg	90.218(1)	93.432(5)	90
γ, deg	90	97.036(5)	120
V, Å^3	20319.6(5)	4655.7(11)	17640.1(18)
T, K	193(2)	193(2)	193(2)
Z	4	2	6
ρ_{calc}, g cm^{-3}	1.326	1.557	1.004
μ, mm^{-1} (MoKα)	4.28	4.547	0.59
F(000)	7992	2188	5436
absorption corrections	multi-scan, 0.59-0.75	multi-scan, 0.65-0.75	multi-scan, 0.66-0.75
θ range, deg	1.22 – 25.25	2.09 – 30.70	1.22 – 25.06
no. of rflns measd	63923	398015	90510
R_{int}	0.068	0.107	0.056
no. of rflns unique	63923	28565	13863
no. of params / restraints	2886 / 4241	1086 / 748	616 / 2
R_1 ($I > 2\sigma(I)$) [a]	0.072	0.043	0.046
R_1 (all data)	0.095	0.089	0.067
wR_2 ($I > 2\sigma(I)$)	0.199	0.096	0.129
wR_2 (all data)	0.218	0.123	0.151
Diff.Fourier.peaks min/max, eÅ^{-3}	-1.342 / 3.324	-2.452 / 3.306	-0.514 / 0.554
CCDC number	1973686	1973691	1973685

2. NMR spectra of new compound

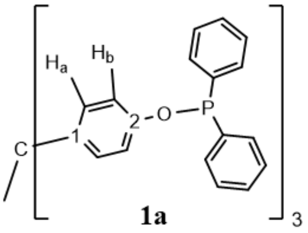

^{1}H NMR (CDCl$_3$, 400 MHz, 25ºC)

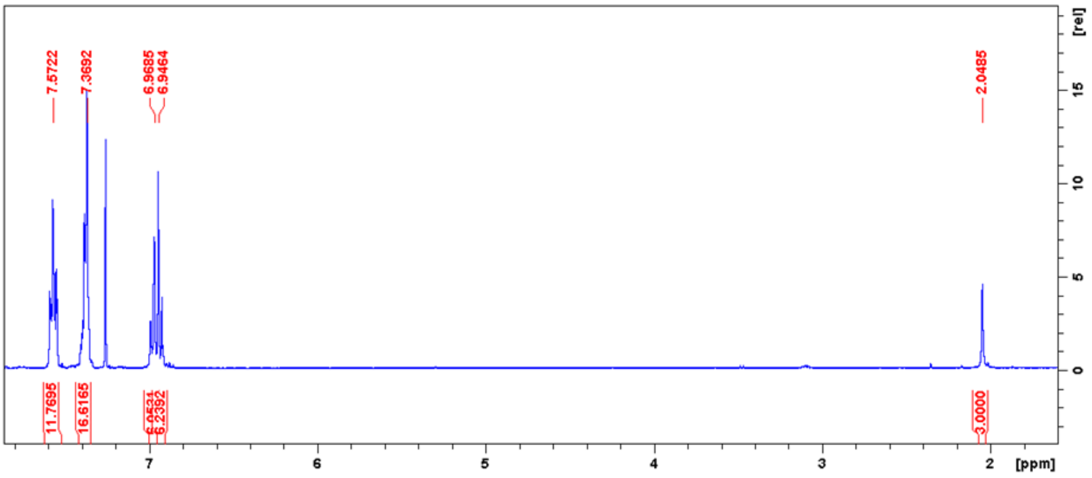

Figure S1. ^{1}H NMR (CDCl$_3$, 400 MHz, 25ºC) of **1a**.

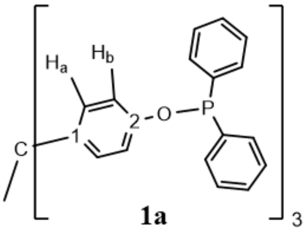

^{31}P {^{1}H} NMR (CDCl$_3$, 162 MHz, 25ºC)

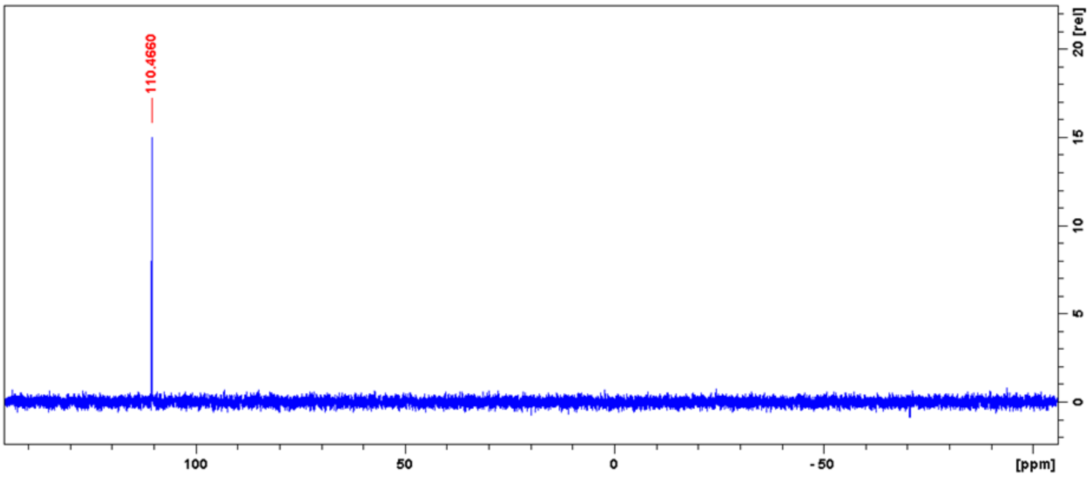

Figure S2. ^{31}P {^{1}H} NMR (CDCl$_3$, 162 MHz, 25ºC) of **1a.**

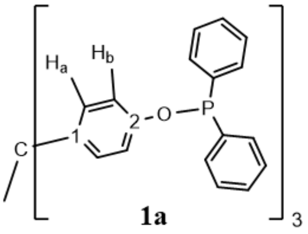

$^{13}C\ \{^1H\}$ NMR
($CDCl_3$, 100 MHz,
25ºC)

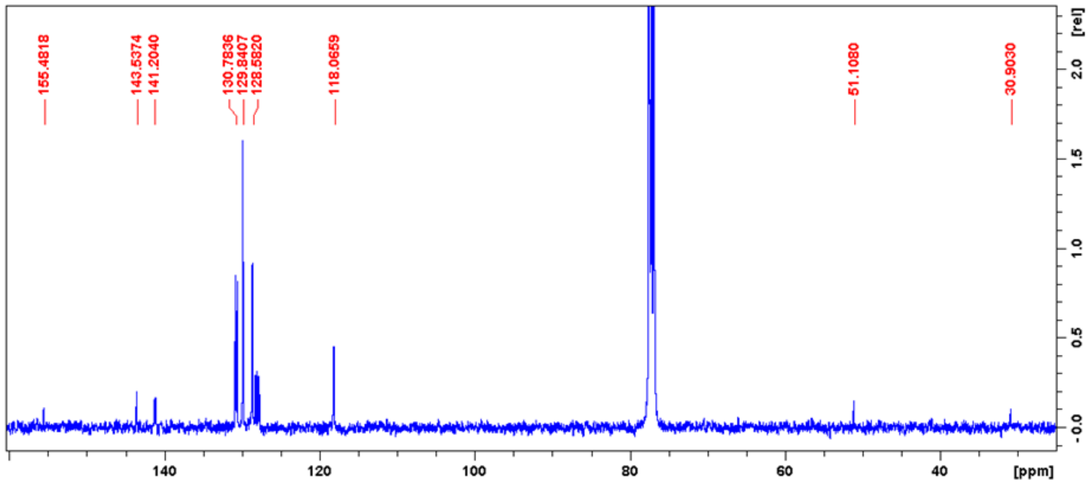

Figure S3. $^{13}C\ \{^1H\}$ NMR ($CDCl_3$, 100 MHz, 25ºC) of **1a**.

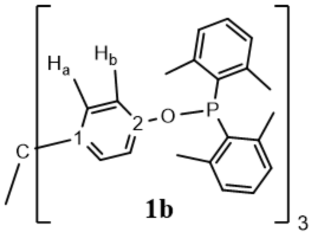

^1H NMR
($CDCl_3$, 400 MHz,
25ºC)

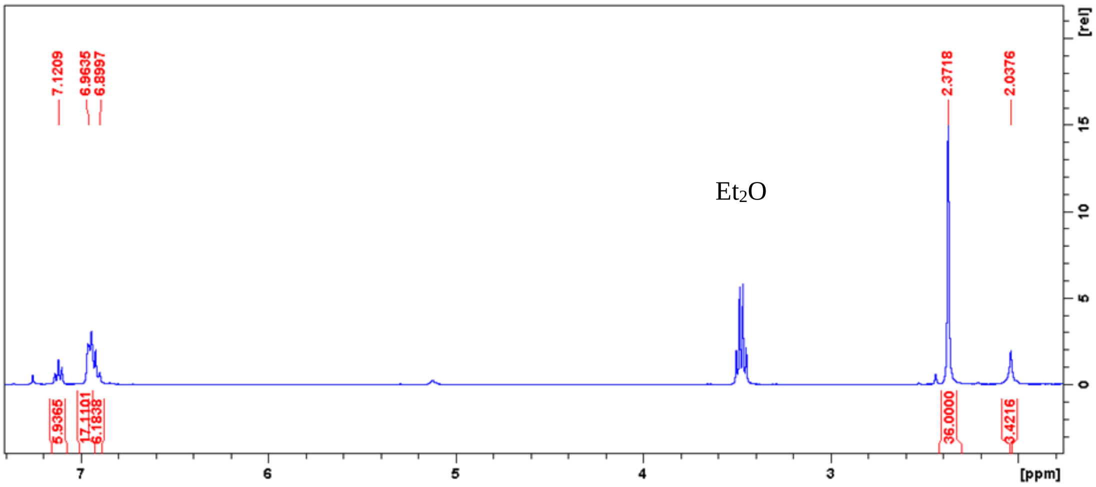

Figure S4. ^1H NMR ($CDCl_3$, 400 MHz, 25ºC) of **1b**.

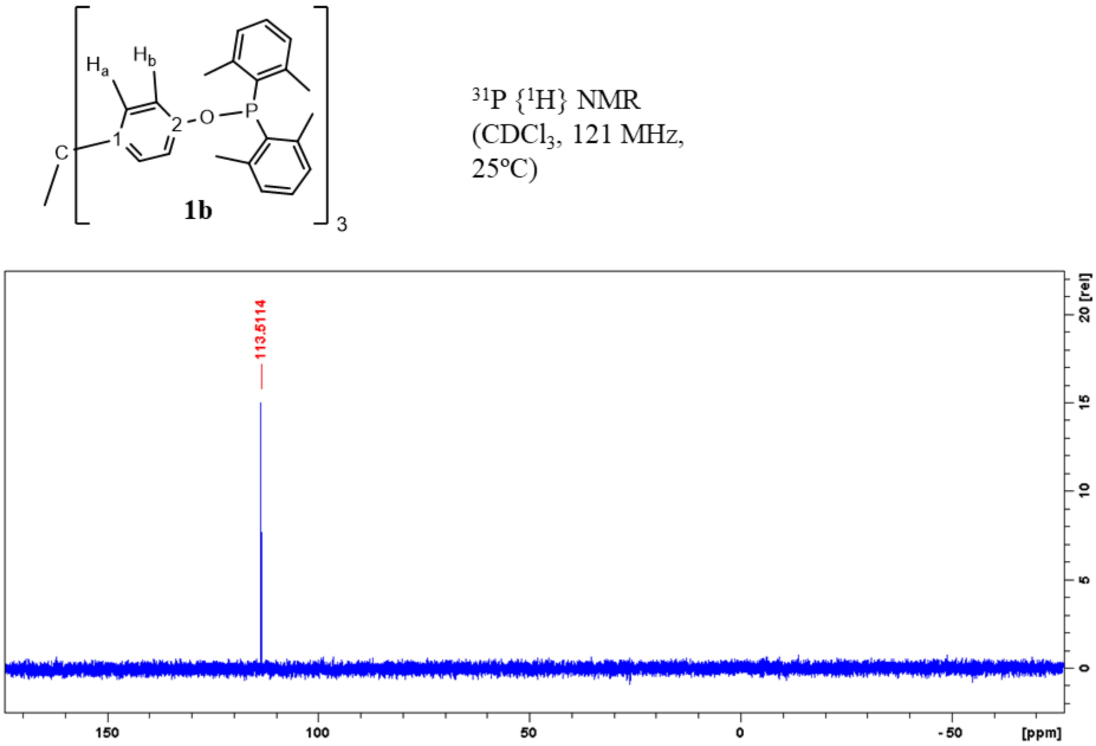

Figure S5. ^{31}P {^{1}H} NMR ($CDCl_3$, 121 MHz, 25ºC) of **1b**.

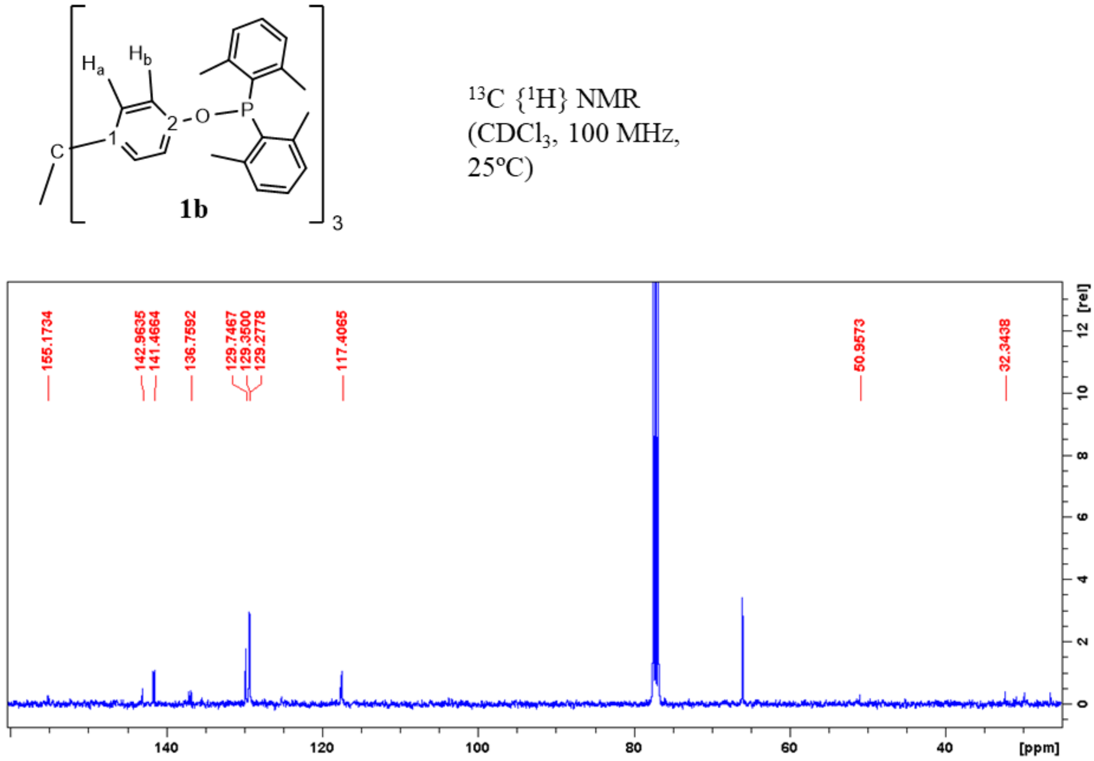

Figure S6. ^{13}C {^{1}H} NMR ($CDCl_3$, 100 MHz, 25ºC) of **1b**.

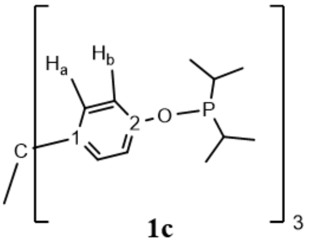

^{1}H NMR
($CDCl_3$, 300 MHz,
25ºC)

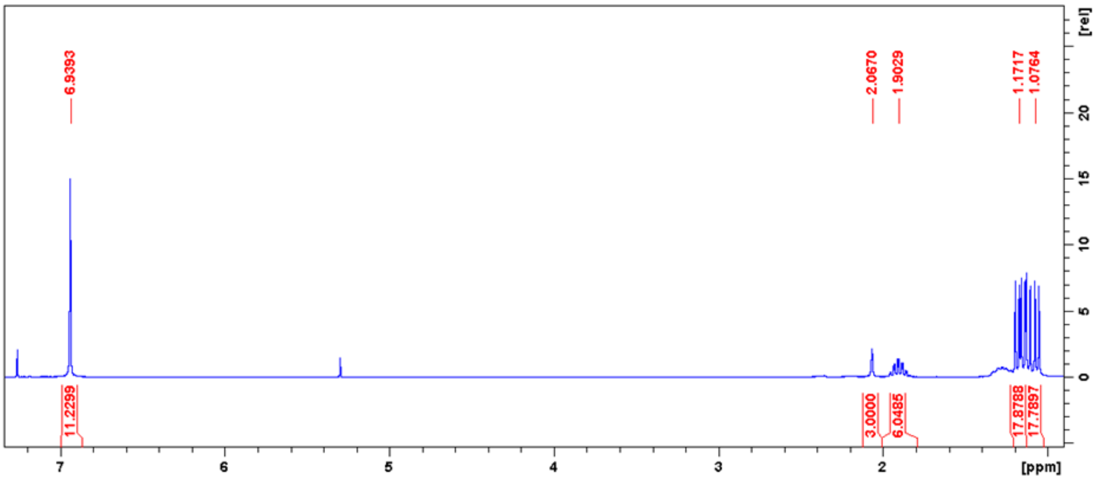

Figure S7. ^{1}H NMR ($CDCl_3$, 300 MHz, 25ºC) of **1c**.

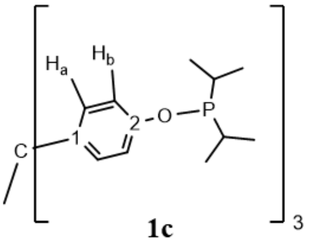

^{31}P {^{1}H} NMR
($CDCl_3$, 121 MHz,
25ºC)

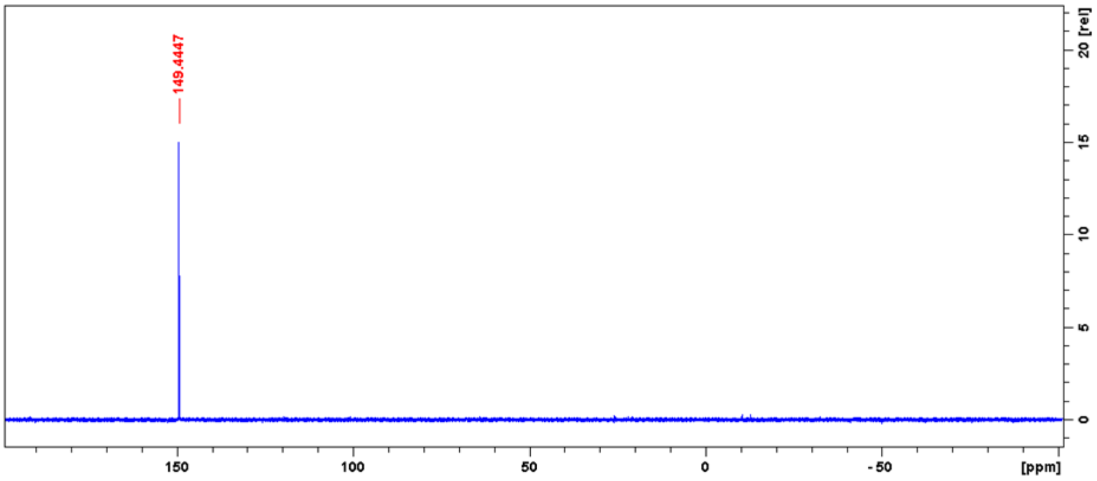

Figure S8. ^{31}P {^{1}H} NMR ($CDCl_3$, 121 MHz, 25ºC) of **1c**.

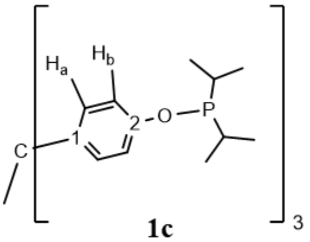

1c

^{13}C {^{1}H} NMR
($CDCl_3$, 75 MHz,
25ºC)

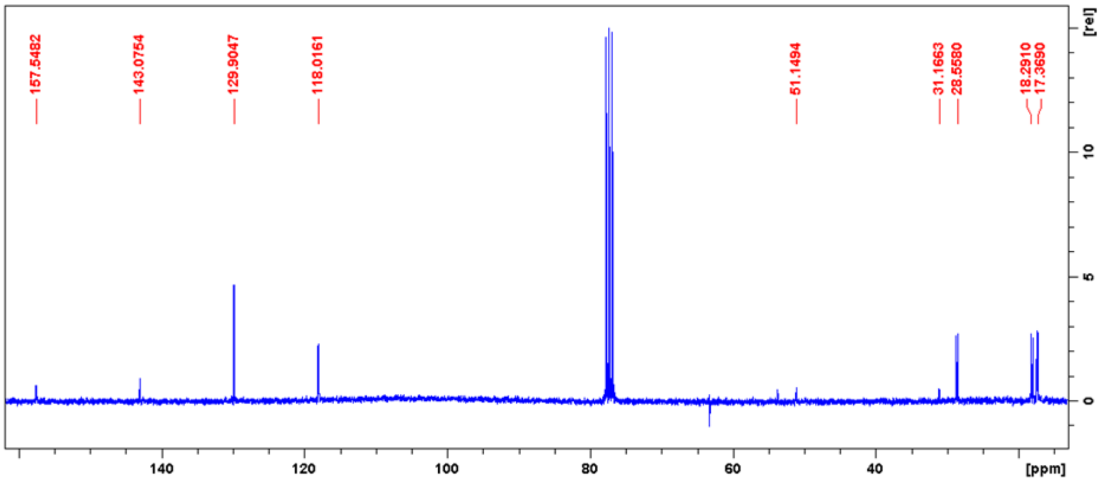

Figure S9. ^{13}C {^{1}H} NMR ($CDCl_3$, 75 MHz, 25ºC) of **1c**.

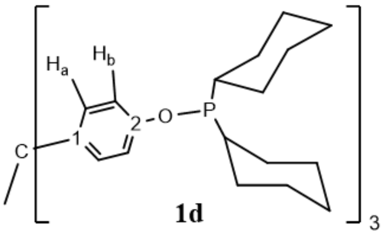

1d

^{1}H NMR
(CD_2Cl_2, 400 MHz,
25ºC)

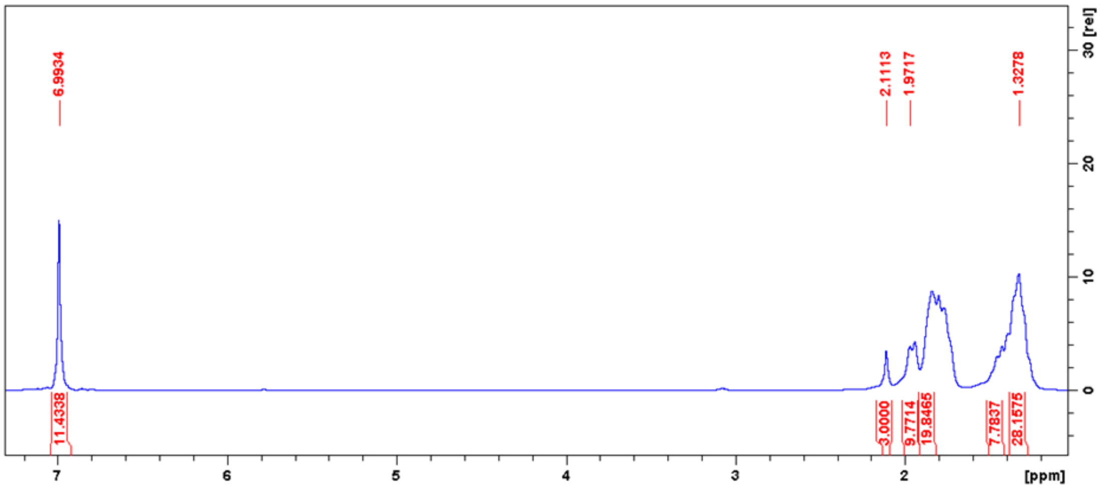

Figure S10. ^{1}H NMR (CD_2Cl_2, 400 MHz, 25ºC) of **1d**.

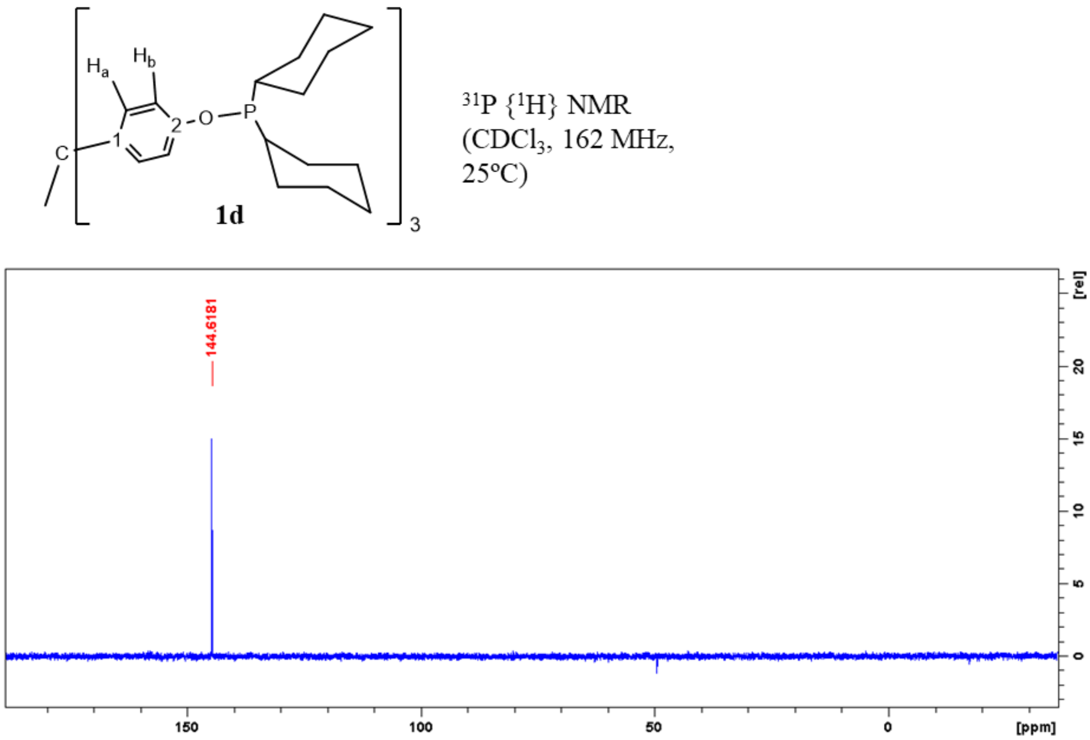

Figure S11. ^{31}P {^{1}H} NMR (CD_2Cl_2, 162 MHz, 25ºC) of **1d**.

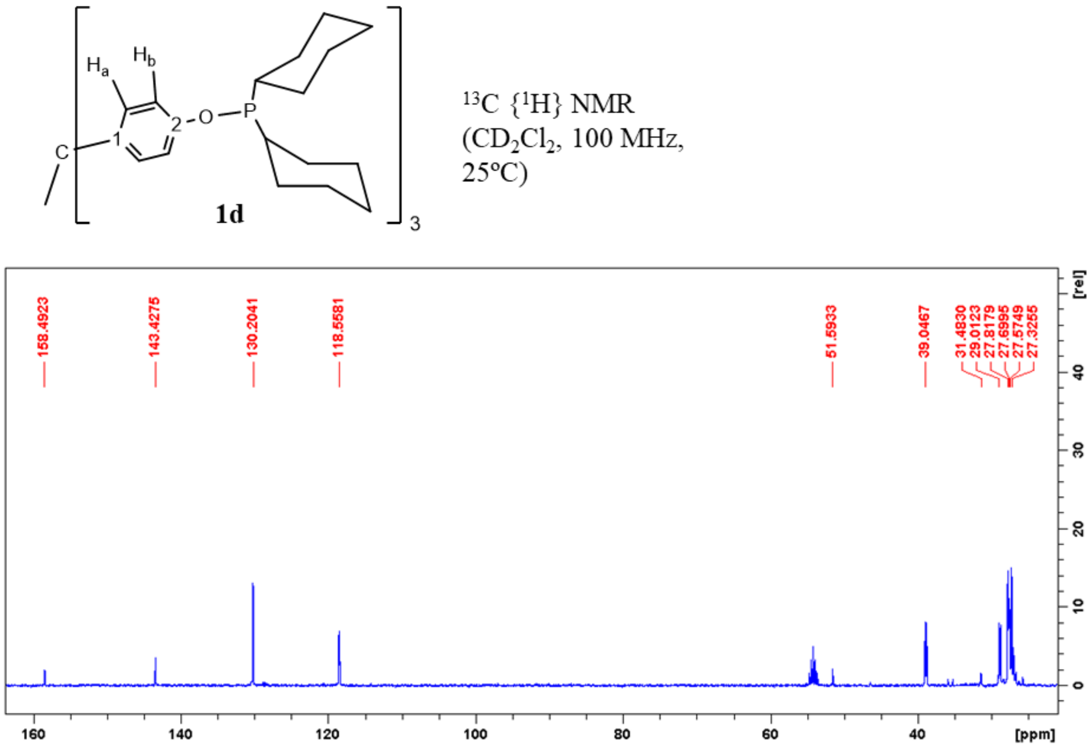

Figure S12. ^{13}C {^{1}H} NMR (CD_2Cl_2, 100 MHz, 25ºC) of **1d**.

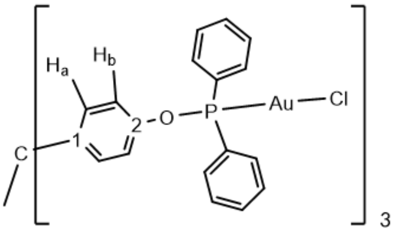

2a

^{1}H NMR
(CD_2Cl_2, 400 MHz,
25ºC)

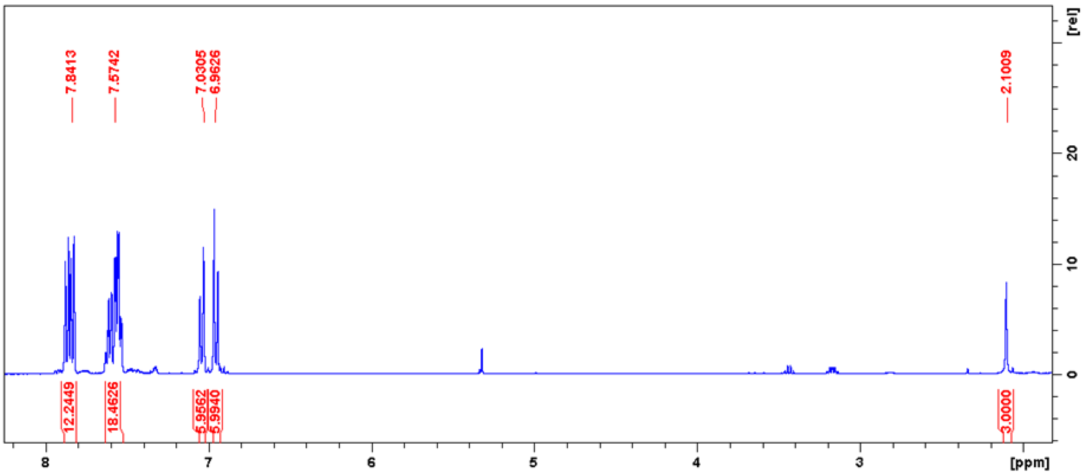

Figure S13. ^{1}H NMR (CD_2Cl_2, 400 MHz, 25ºC) of **2a**.

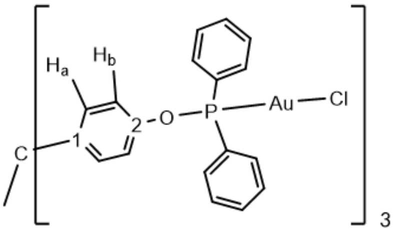

2a

^{31}P {^{1}H} NMR
(CD_2Cl_2, 162 MHz,
25ºC)

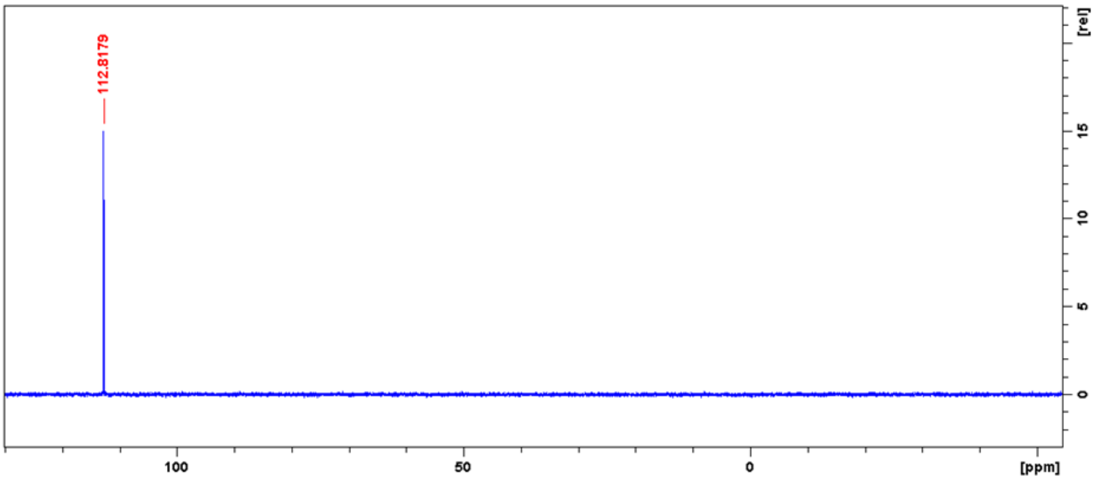

Figure S14. ^{31}P {^{1}H} NMR (CD_2Cl_2, 162 MHz, 25ºC) of **2a**.

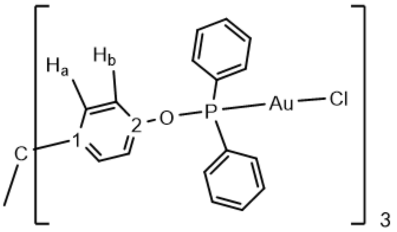

2a

^{13}C {^{1}H} NMR
(CD_2Cl_2, 100 MHz,
25ºC)

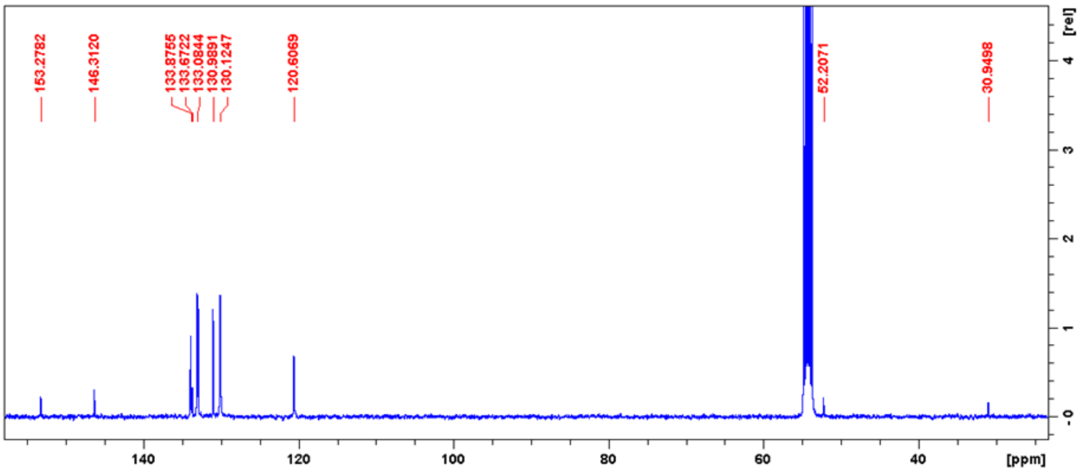

Figure S15. ^{13}C {^{1}H} NMR (CD_2Cl_2, 100 MHz, 25ºC) of **2a**.

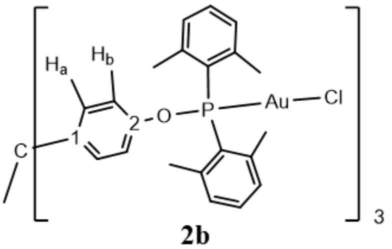

2b

^{1}H NMR
(CD_2Cl_2, 400 MHz,
25ºC)

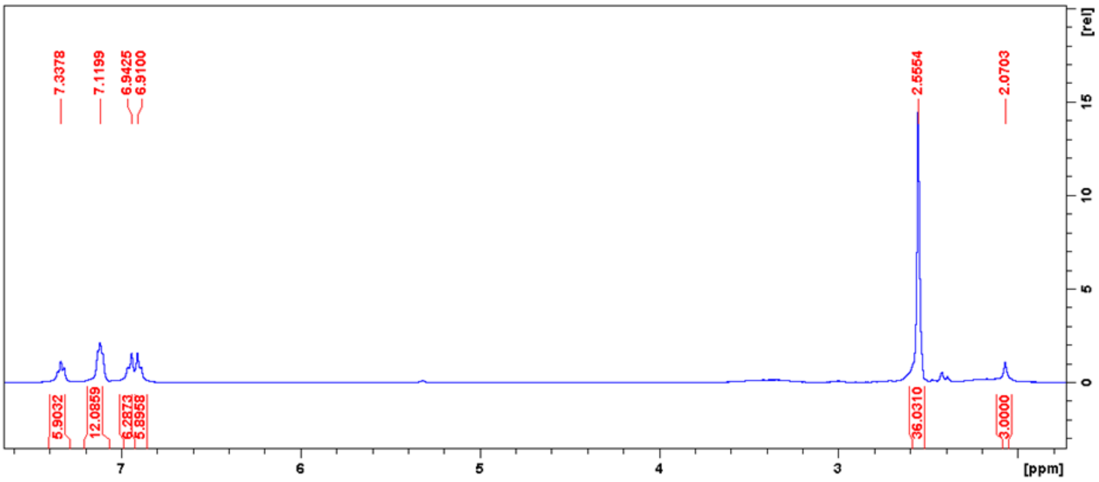

Figure S16. ^{1}H NMR (CD_2Cl_2, 400 MHz, 25ºC) of **2b**.

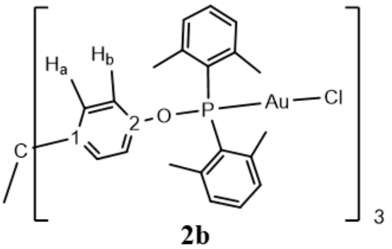

2b

^{31}P {^1H} NMR (CD_2Cl_2, 202 MHz, 25ºC)

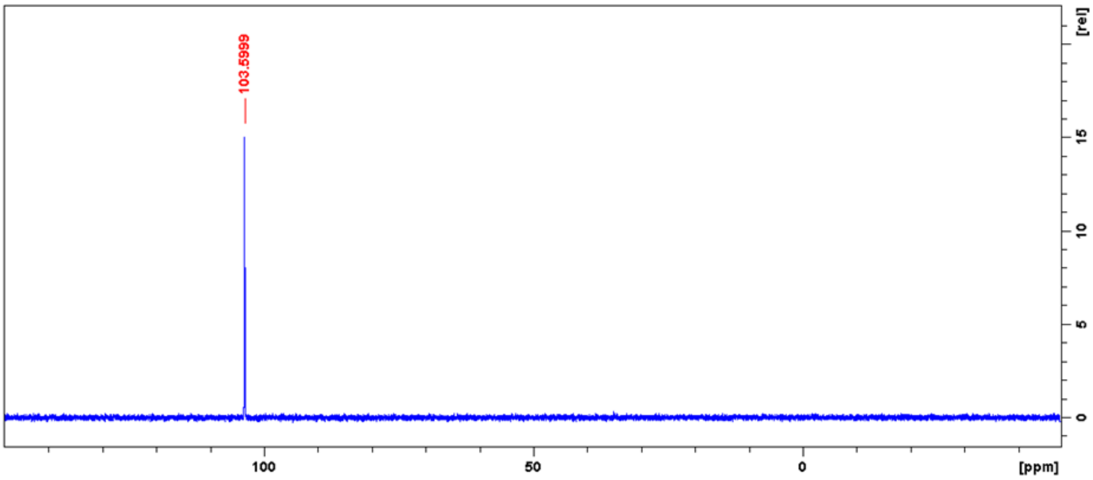

Figure S17. ^{31}P {^1H} NMR (CD_2Cl_2, 202 MHz, 25ºC) of **2b**.

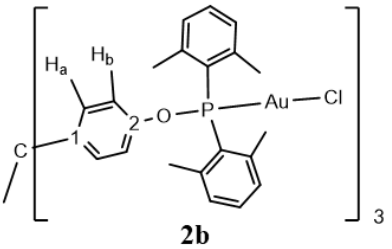

2b

^{13}C {^1H} NMR (CD_2Cl_2, 100 MHz, 25ºC)

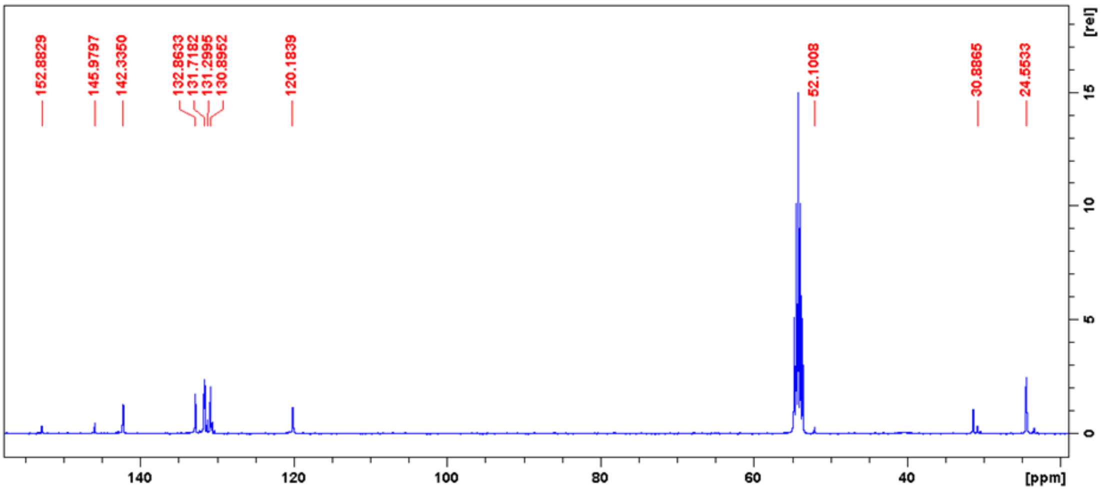

Figure S18. ^{13}C {^1H} NMR (CD_2Cl_2, 100 MHz, 25ºC) of **2b**.

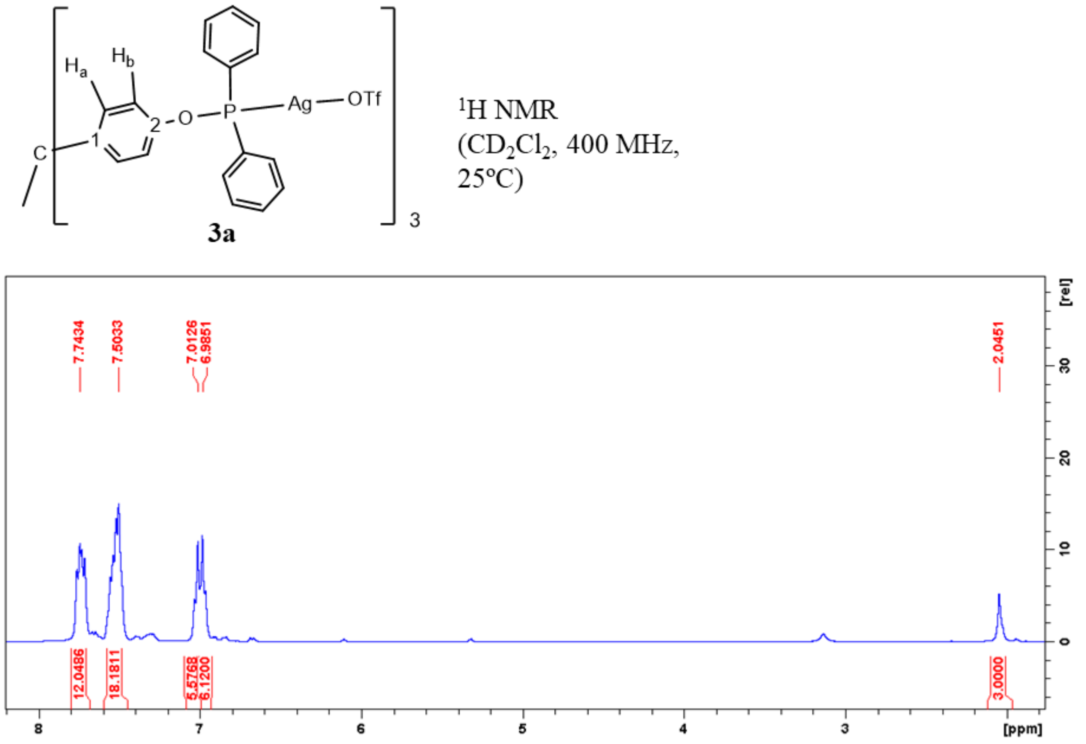

Figure S19. ^{1}H NMR (CD_2Cl_2, 400 MHz, 25ºC) of **3a**.

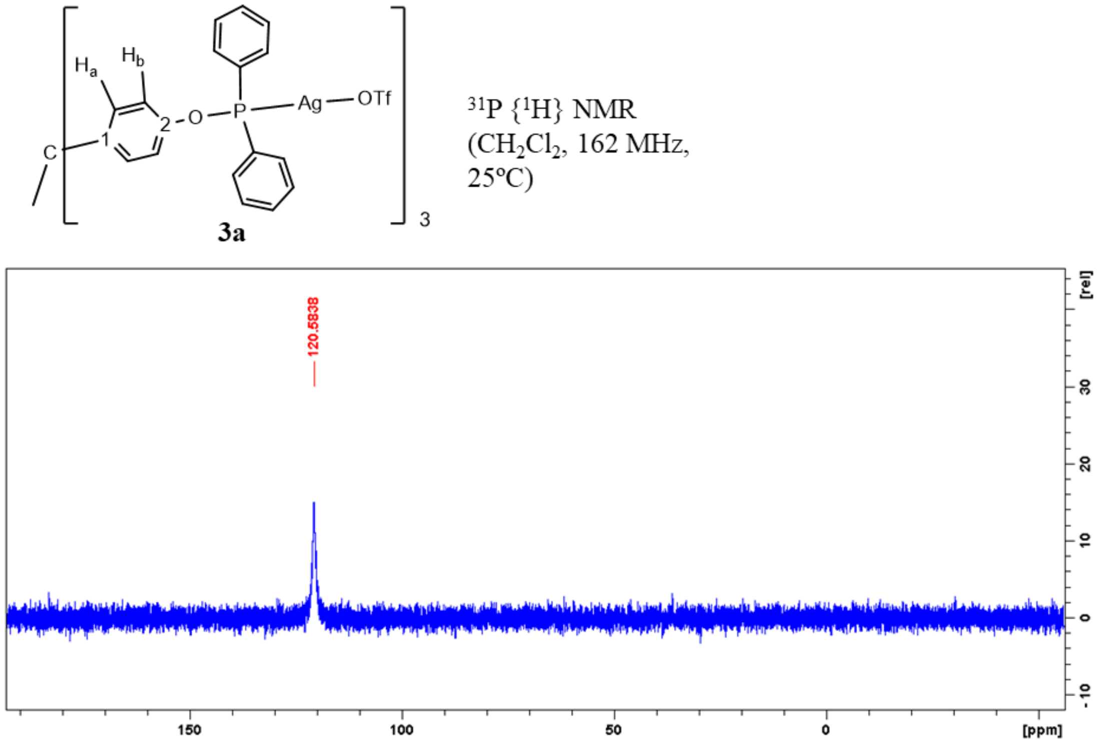

Figure S20. ^{31}P {^{1}H} NMR (CD_2Cl_2, 162 MHz, 25ºC) of **3a**.

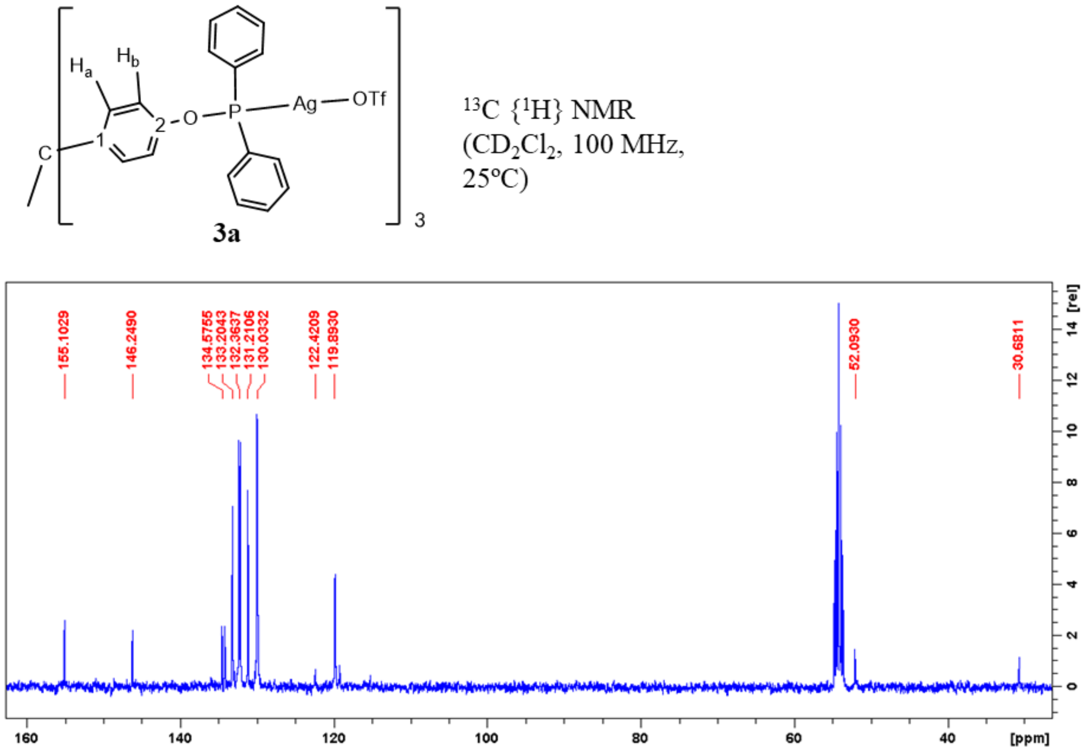

Figure S21. ^{13}C {^{1}H} NMR (CD_2Cl_2, 100 MHz, 25ºC) of **3a**.

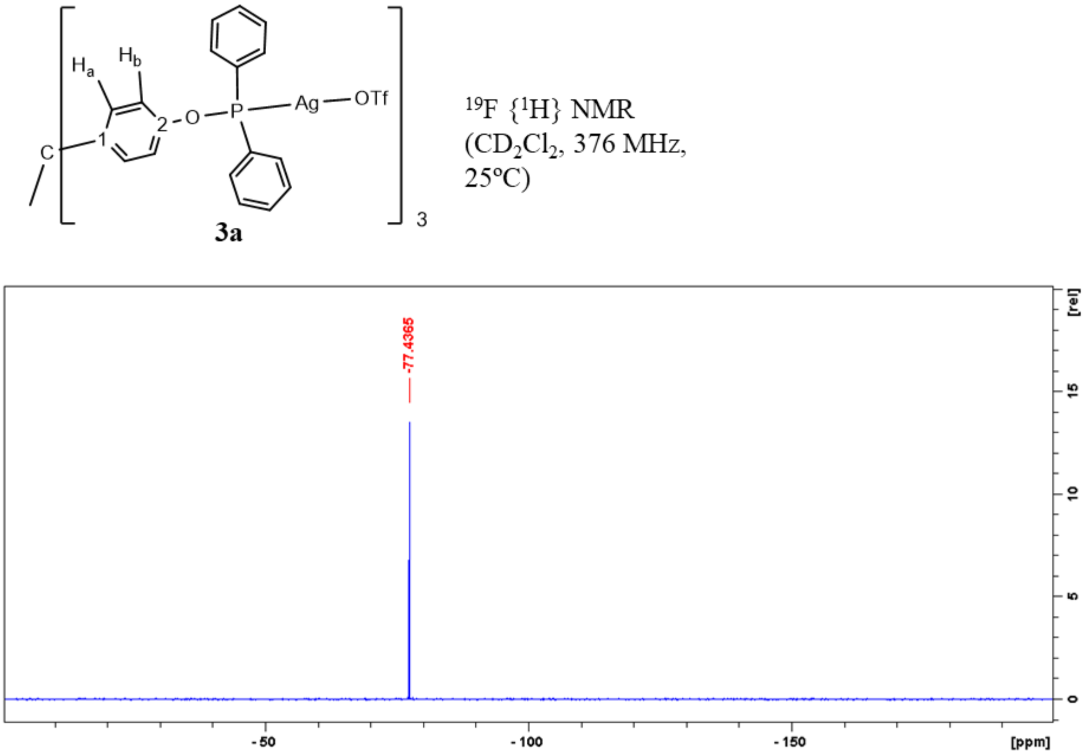

Figure S22. ^{19}F {^{1}H} NMR (CD_2Cl_2, 376 MHz, 25ºC) of **3a**.

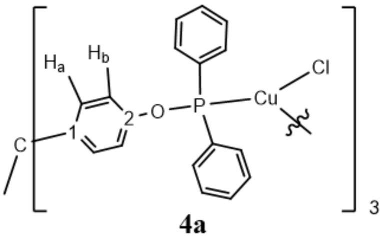

4a

^{1}H NMR
($CDCl_3$, 400 MHz,
25ºC)

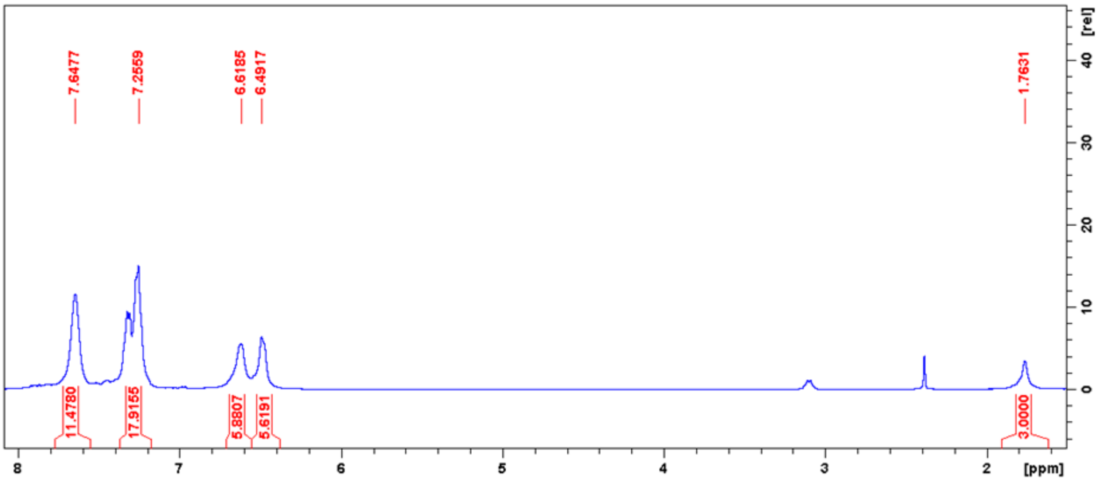

Figure S23. ^{1}H NMR ($CDCl_3$, 400 MHz, 25ºC) of **4a**.

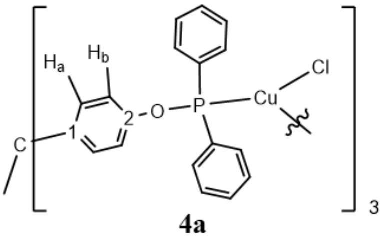

4a

^{31}P {^{1}H} NMR
($CDCl_3$, 162 MHz,
25ºC)

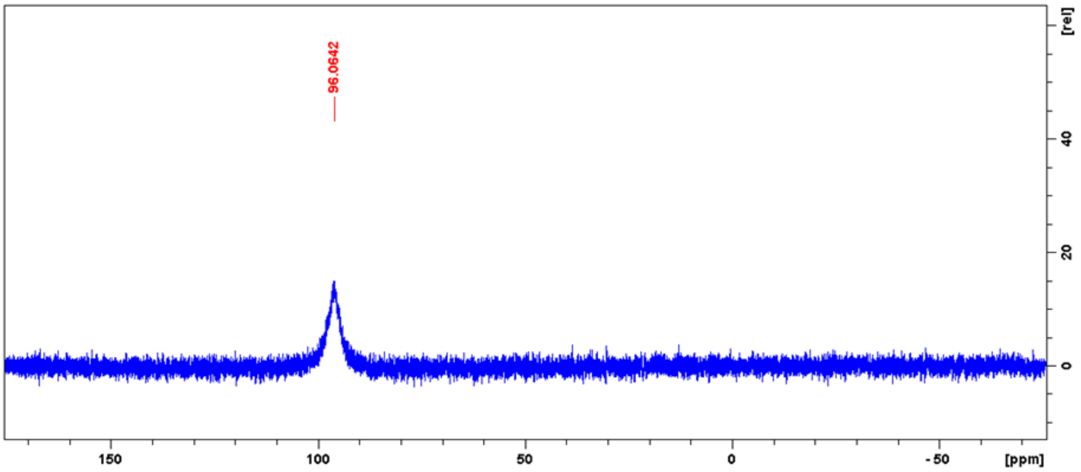

Figure S24. ^{31}P {^{1}H} NMR ($CDCl_3$, 162 MHz, 25ºC) of **4a**.

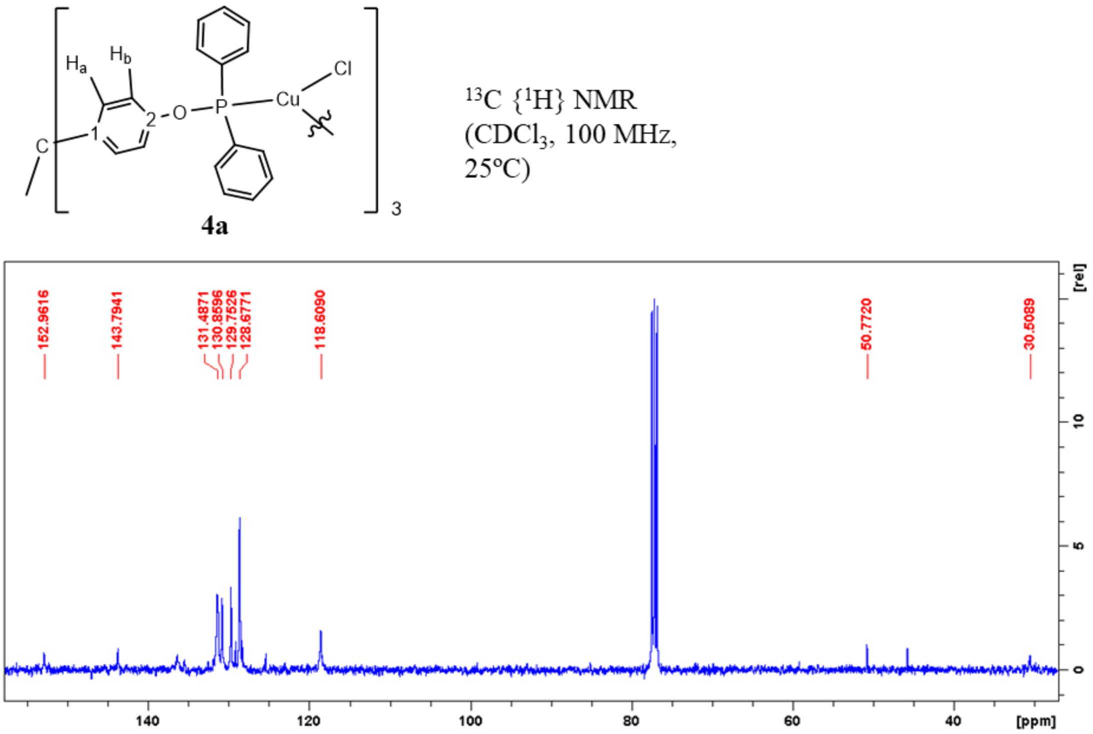

Figure S25. ^{13}C {^{1}H} NMR ($CDCl_3$, 100 MHz, 25ºC) of **4a**.

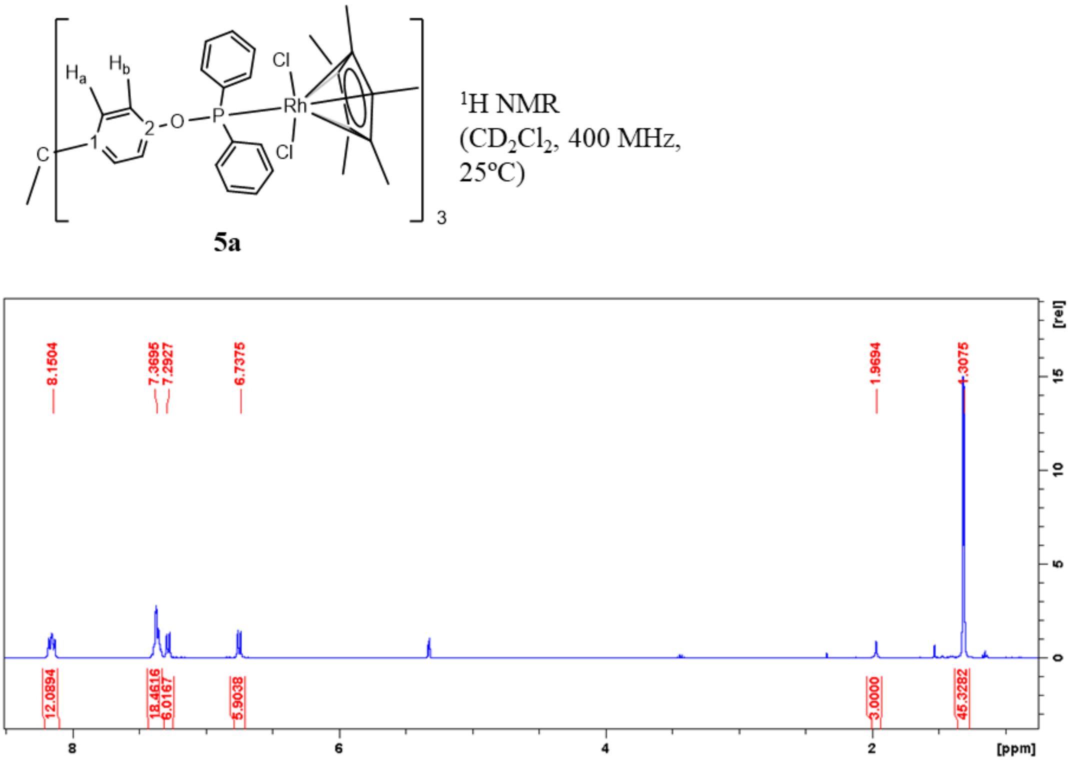

Figure S26. ^{1}H NMR (CD_2Cl_2, 400 MHz, 25ºC) of **5a**.

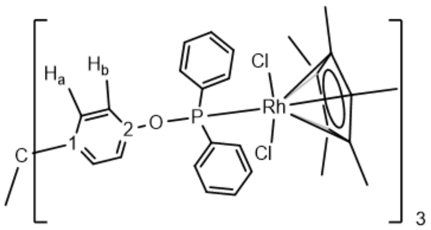

5a

^{31}P {^{1}H} NMR (CD_2Cl_2, 121 MHz, 25ºC)

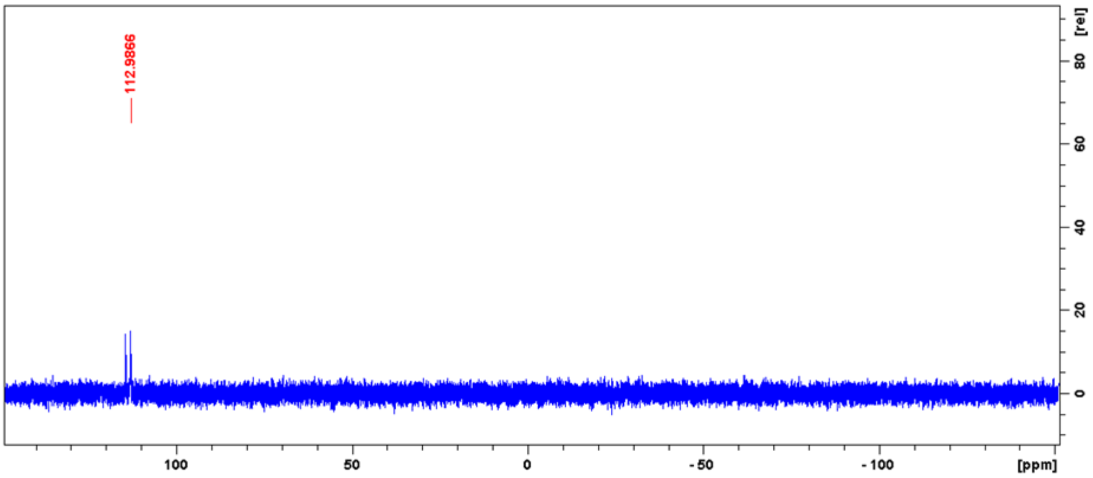

Figure S27. ^{31}P {^{1}H} NMR (CD_2Cl_2, 121 MHz, 25ºC) of **5a**.

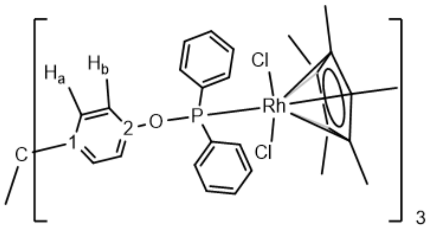

5a

^{13}C {^{1}H} NMR (CD_2Cl_2, 100 MHz, 25ºC)

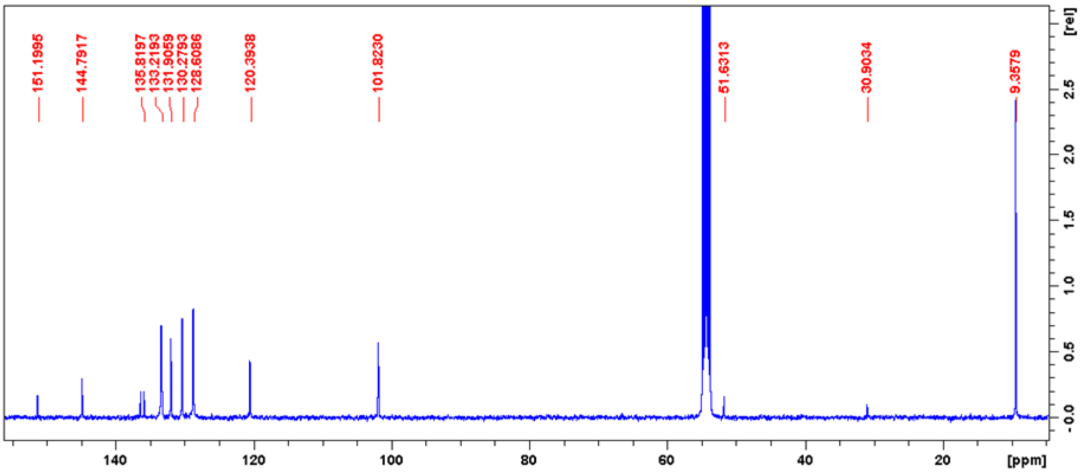

Figure S28. ^{13}C {^{1}H} NMR (CD_2Cl_2, 100 MHz, 25ºC) of **5a**.

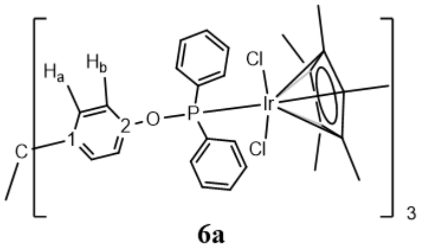

6a

^{1}H NMR (CD_2Cl_2, 400 MHz, 25ºC)

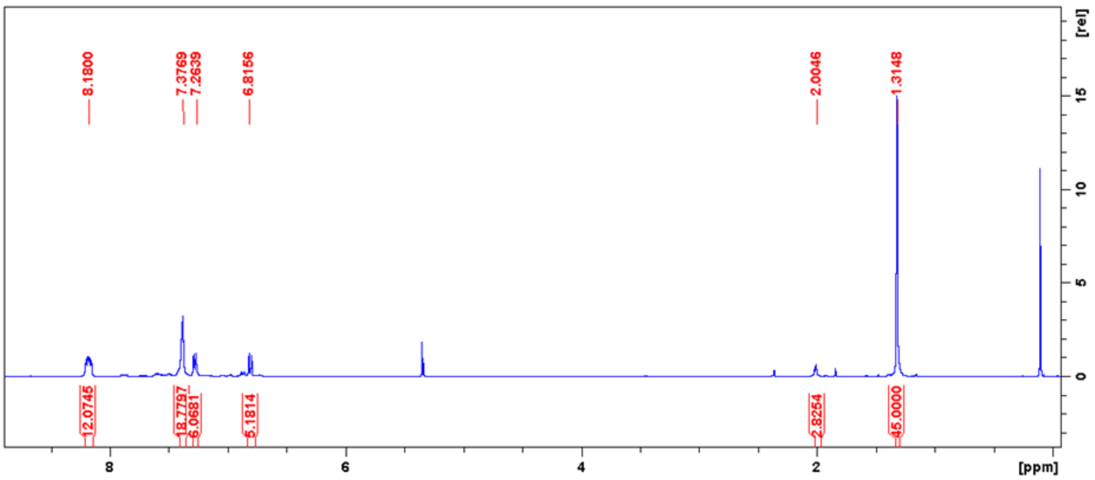

Figure S29. ^{1}H NMR (CD_2Cl_2, 400 MHz, 25ºC) of **6a**.

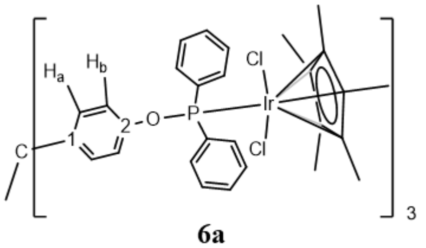

6a

^{31}P {^{1}H} NMR (CD_2Cl_2, 121 MHz, 25ºC)

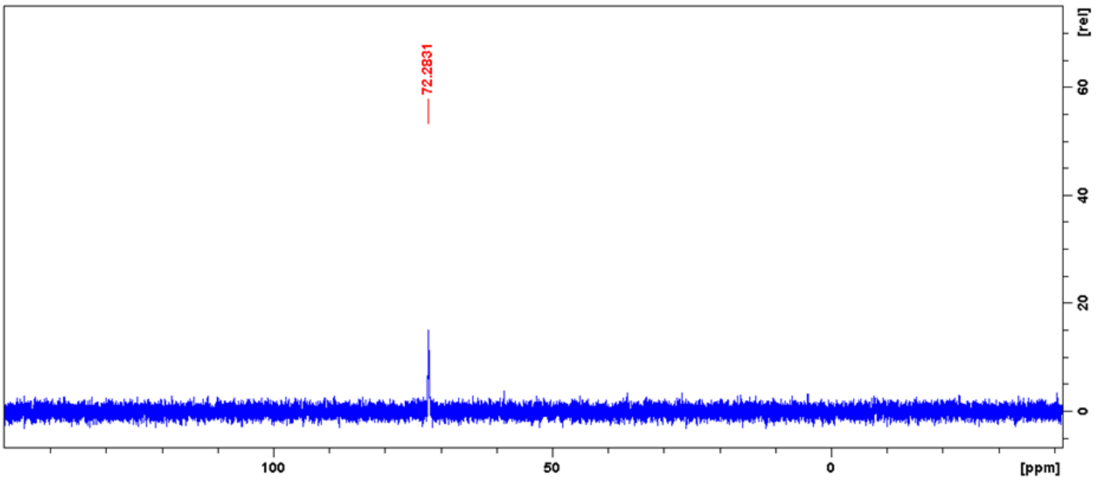

Figure S30. ^{31}P {^{1}H} NMR (CD_2Cl_2, 121 MHz, 25ºC) of **6a**.

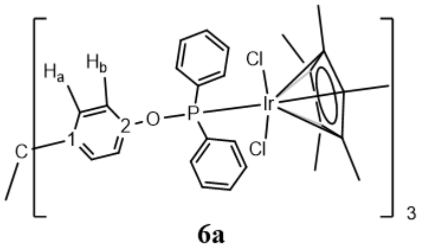

6a

$^{13}C\ \{^{1}H\}$ NMR (CD_2Cl_2, 100 MHz, 25ºC)

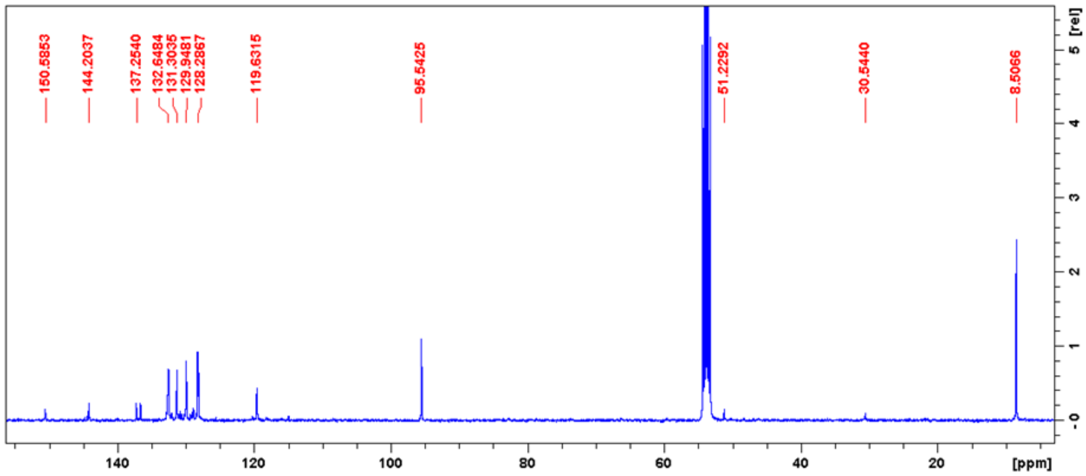

Figure S31. $^{13}C\ \{^{1}H\}$ NMR (CD_2Cl_2, 100 MHz, 25ºC) of **6a**.

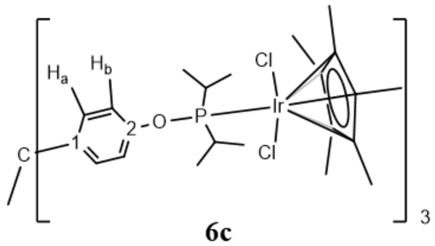

6c

^{1}H NMR (CD_2Cl_2, 400 MHz, 25ºC)

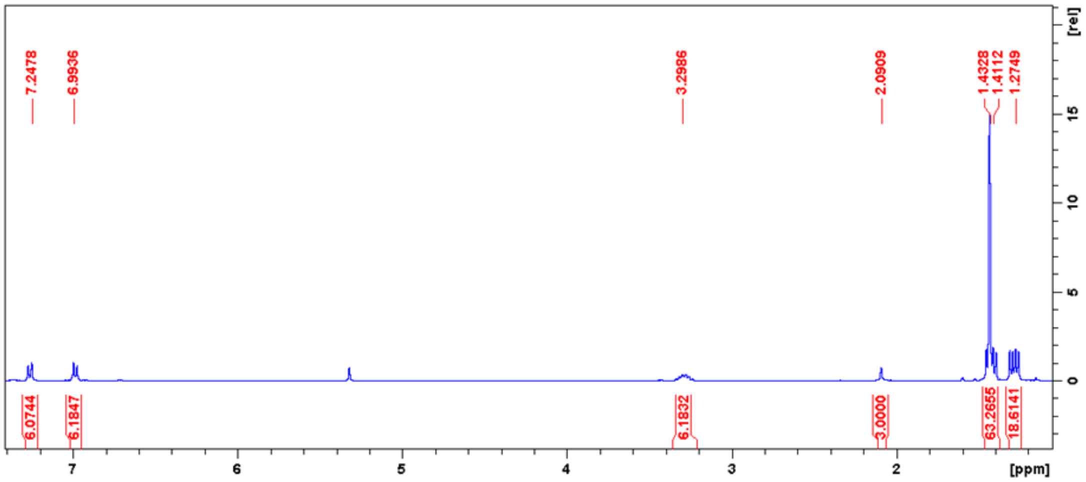

Figure S32. ^{1}H NMR (CD_2Cl_2, 400 MHz, 25ºC) of **6c**.

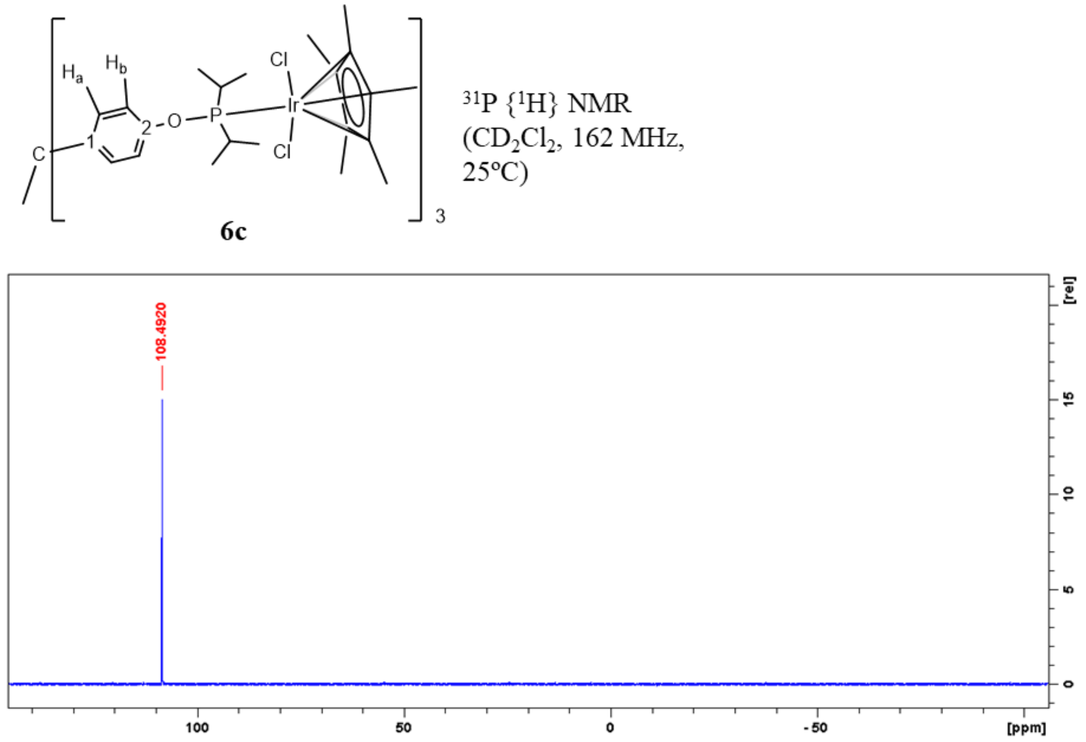

Figure S33. ^{31}P {^{1}H} NMR (CD_2Cl_2, 162 MHz, 25ºC) of **6c**.

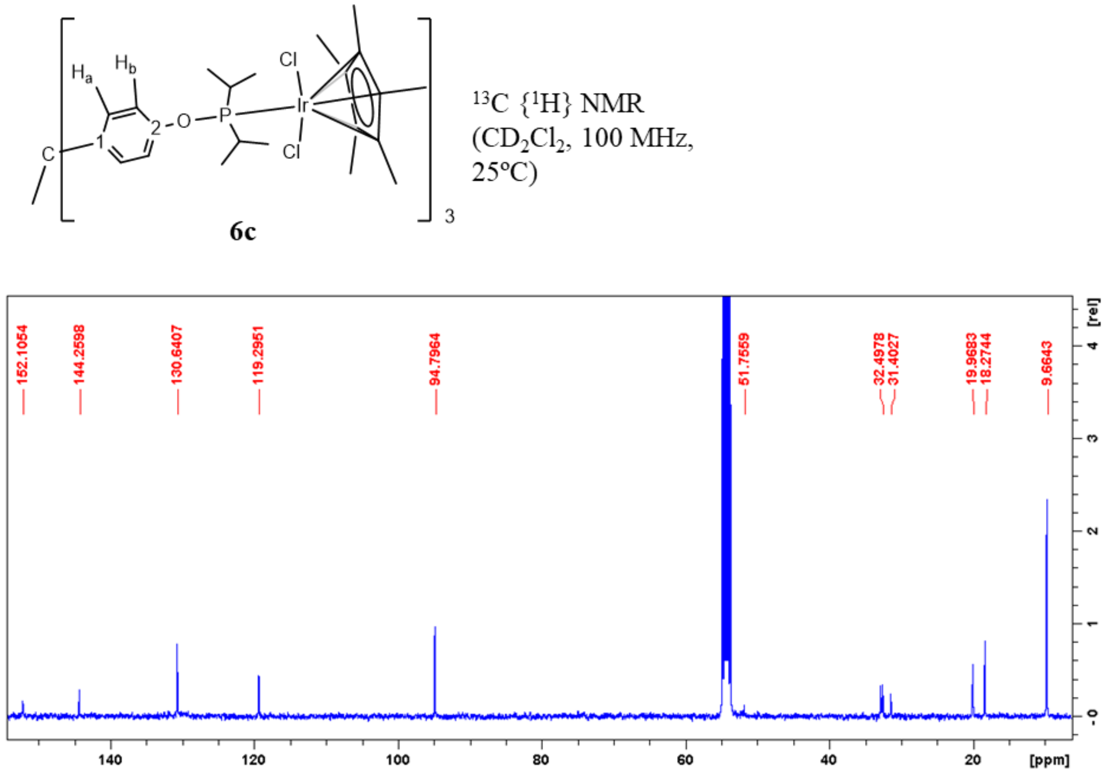

Figure S34. ^{13}C {^{1}H} NMR (CD_2Cl_2, 100 MHz, 25ºC) of **6c**.

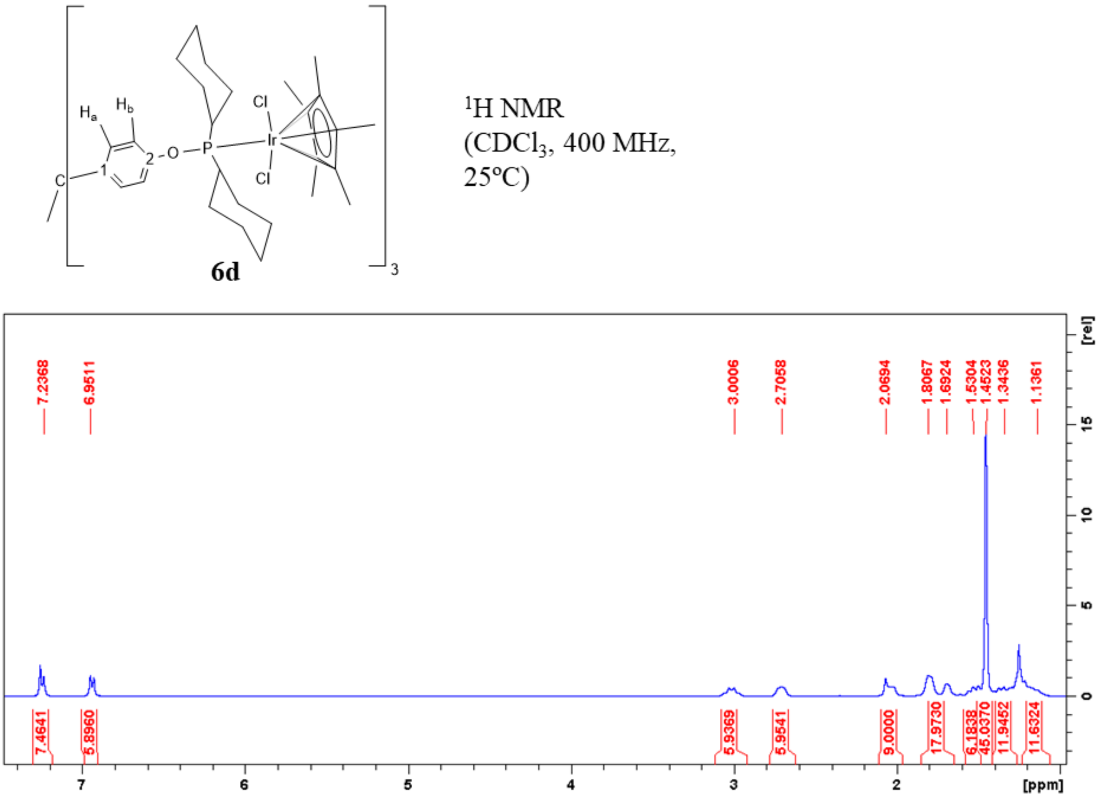

Figure S35. ^{1}H NMR ($CDCl_3$, 400 MHz, 25ºC) of **6d**.

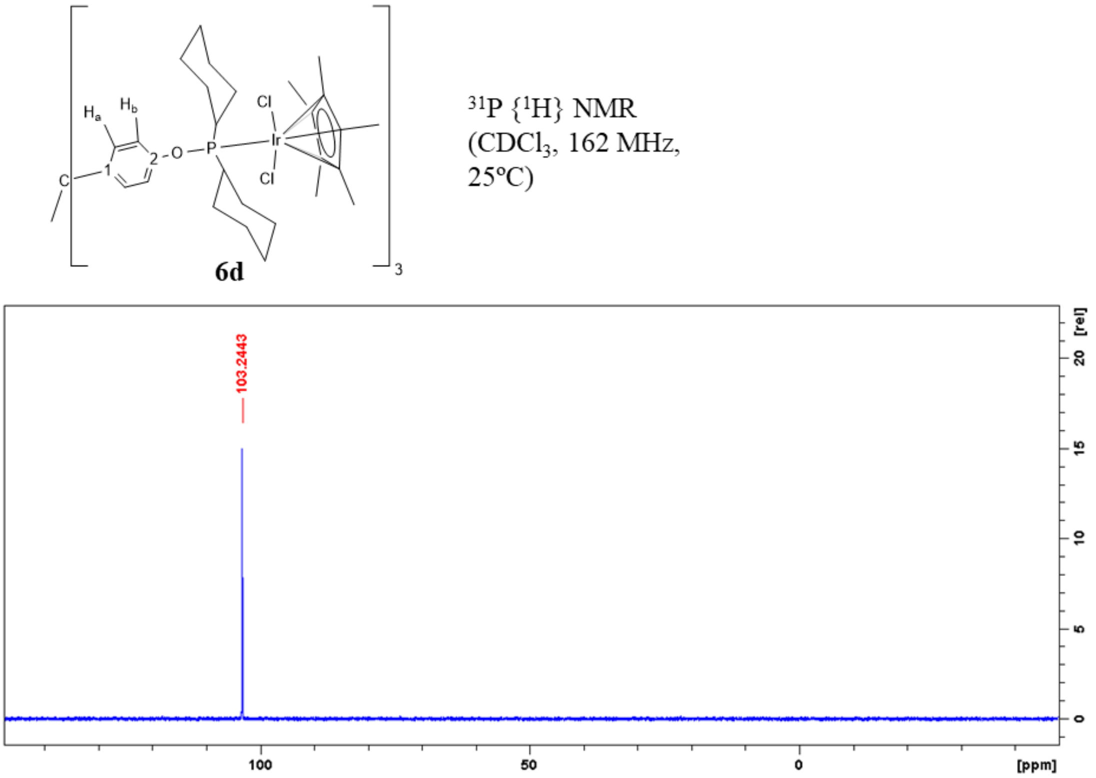

Figure S36. ^{31}P {^{1}H} NMR ($CDCl_3$, 162 MHz, 25ºC) of **6d**.

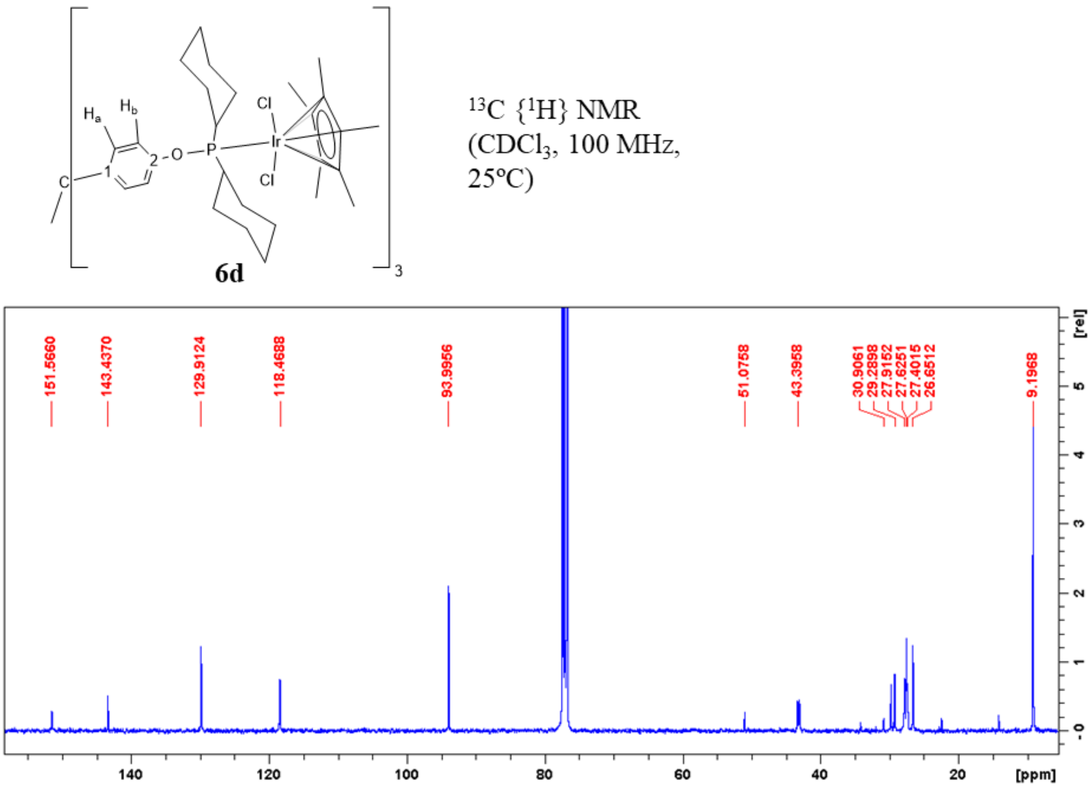

Figure S37. ^{13}C {^{1}H} NMR ($CDCl_3$, 100 MHz, 25ºC) of **6d**.

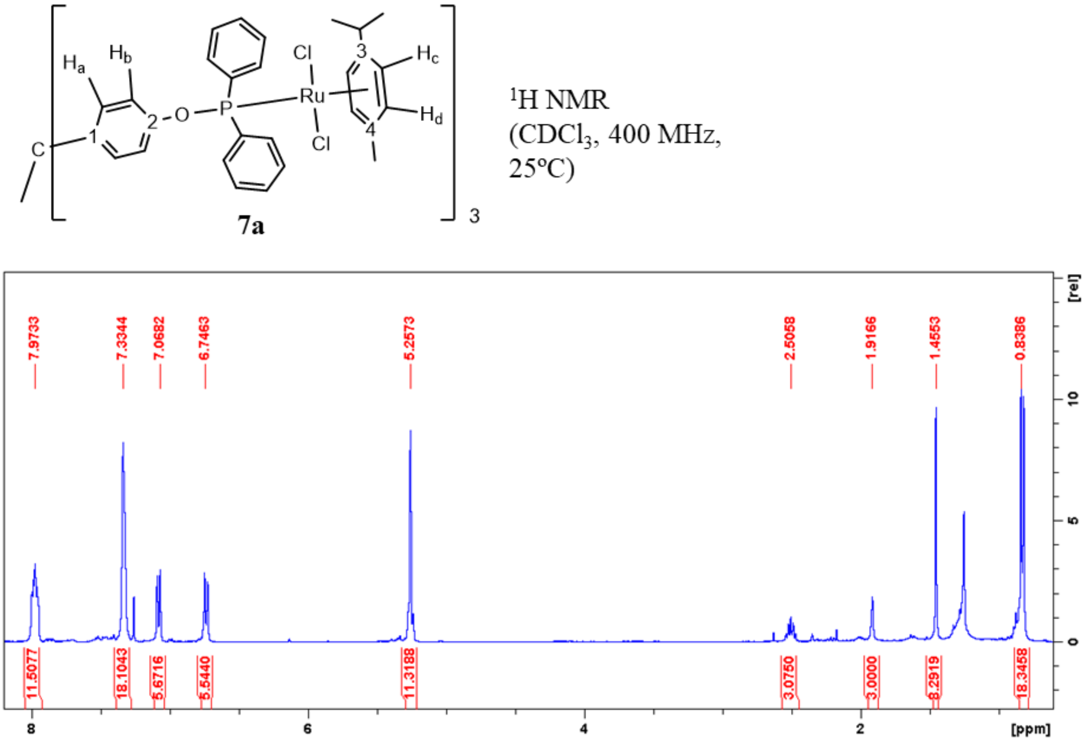

Figure S38. ^{1}H NMR ($CDCl_3$, 400 MHz, 25ºC) of **7a**.

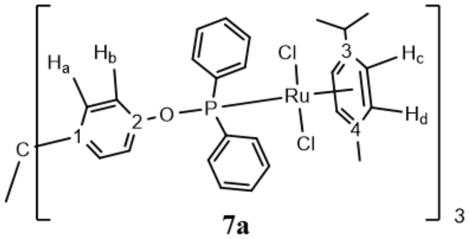

7a

^{31}P {^{1}H} NMR (CDCl$_3$, 121 MHz, 25ºC)

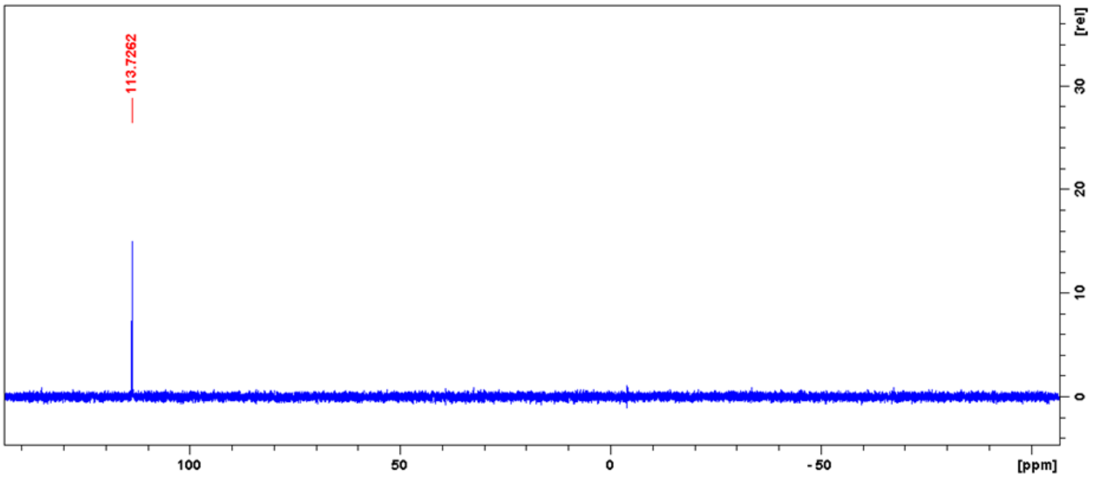

Figure S39. ^{31}P {^{1}H} NMR (CDCl$_3$, 121 MHz, 25ºC) of **7a**.

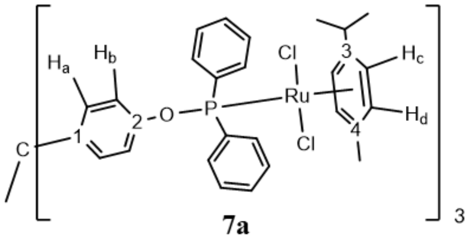

7a

^{13}C {^{1}H} NMR (CDCl$_3$, 100 MHz, 25ºC)

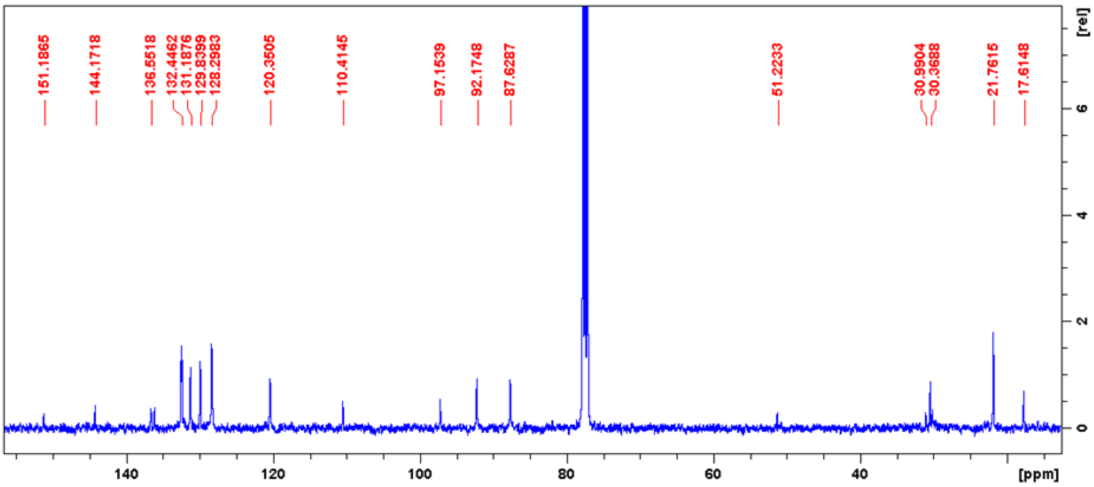

Figure S40. ^{13}C {^{1}H} NMR (CDCl$_3$, 100 MHz, 25ºC) of **7a**.

3. MS (ESI) spectrum of compound 6a

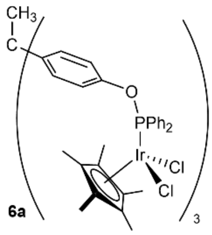

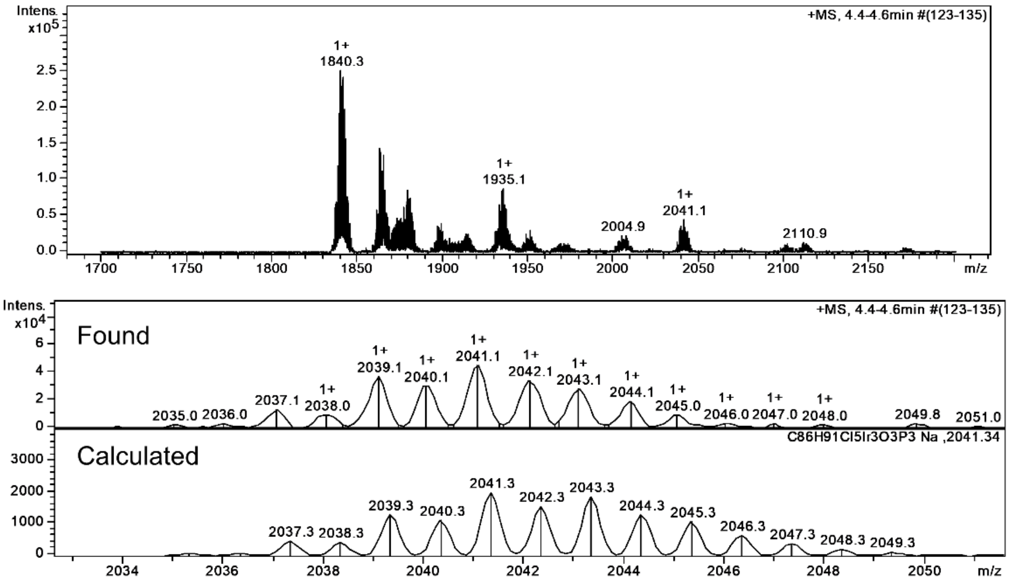

Figure S41. MS (ESI) of **6a**.

4. References

[1] Sheldrick GM. SADABS, Program for Empirical Absorption Correction of Area Detector Data. Göttingen: University of Göttingen; 1996.
[2] G. M. Sheldrick, SHELXTL, version 6.14. Program for solution and refinement of crystal structures, Universität Göttingen, Germany, 2000.
[3] A. L. Spek, *Acta Crystallogr.* **2015**, C71, 9 – 18.
